# Mind the Gap: From Policy to Practice at CNR An Analysis of Career Advancement Criteria in Light of the Agreement on Reforming Research Assessment

**DOI:** 10.12688/openreseurope.23373.1

**Published:** 2026-04-19

**Authors:** Francesca Di Donato, Andrea Mannocci, Fabrizio Pecoraro, Lottie Provost

**Affiliations:** 1 Istituto di Linguistica Computazionale Antonio Zampolli Consiglio Nazionale delle Ricerche, Pisa, Tuscany, 56124, Italy; 2 Istituto di Scienza e Tecnologie dell'Informazione Alessandro Faedo Consiglio Nazionale delle Ricerche, Pisa, Tuscany, 56124, Italy; 3 Istituto di Ricerche sulla Popolazione e le Politiche Sociali Consiglio Nazionale delle Ricerche, Rome, Lazio, 00185, Italy

**Keywords:** Responsible Research Assessment, Career Progression, Policy Implementation, CoARA, Research Performing Organisation

## Abstract

**Background:**

This study examines the practical implementation of the Agreement on Reforming Research Assessment within the National Research Council of Italy by analysing the 2023 competitive procedures for the career progression of researchers and technologists. As the institution formally adopted the reform to shift towards a more responsible and qualitative evaluation framework, this research investigates the extent to which these high-level commitments were applied within the calls by the individual evaluation committees.

**Methods:**

A systematic qualitative and quantitative analysis was conducted on 4,090 evaluation criteria established by 90 independent evaluation committees. The criteria were examined to assess their alignment with the four core commitments of the Agreement and the inclusion of Open Science principles.

**Results:**

Results indicate a significant gap between institutional policy and actual practice: most committees do not fully recognise the diversity of research contributions, often undervaluing or excluding non-traditional outputs, such as teaching and evaluation activities, or public and policy engagement. Quantitative and mechanical metrics continue to be widely used in researcher evaluations, with journal-based indicators often used to assess research quality. Regarding Open Science practices, they appeared only sporadically in the assessed criteria.

**Conclusions:**

These findings suggest that traditional evaluation practices persist at the committee level, despite formal institutional reform. The study highlights that high-level policy adoption alone is insufficient without robust guidance to operationalise reform principles during the assessment process. This research provides insights into the challenges of transitioning from policy to practice in research assessment within a large, multidisciplinary Research Performing Organisation.

## Plain Language summary

This study examines how the reform of research evaluation introduced through the Agreement on Reforming Research Assessment (2022) is being implemented at the National Research Council of Italy (CNR). It also proposes a framework to assess whether evaluation practices comply with the core principles of the Agreement.

In recent years, the reform has brought together many research organisations across Europe that have agreed that researchers should be evaluated in a fairer and more responsible way. Instead of focusing mainly on numerical indicators, such as how many articles a researcher publishes or the ranking of the journals where they appear, the reform encourages evaluating researchers based on a wider range of contributions, and to base the assessment on qualitative judgement, only supported by quantitative indicators.

To understand to what extent the principles of the reform are being applied, we analysed the evaluation criteria used in the 2023 promotion calls for researchers and technologists and assessed them against the principles of the Agreement. We also analysed whether Open Science practices were mentioned as part of the assessment criteria. In total, we examined over 4,000 evaluation criteria used across 90 different career advancement fields corresponding to the disciplinary areas defined by CNR.

The study highlights a persistent gap between policy commitments and evaluation practices. Traditional approaches remain common: quantitative indicators are still used as proxies for research quality, while the diversity of contributions to science is often undervalued. By proposing a method for assessing compliance with the reform’s principles, this study contributes to wider discussions about how these changes can be interpreted and implemented in research performing organisations.

## 1. Introduction

This study analyses the implementation of the Agreement on Reforming Research Assessment (ARRA) in the latest competitive procedures for career progression among researchers and technologists at the National Research Council of Italy (CNR).

The ARRA core commitments - which we report below in Table 1, for the reader’s convenience - outline four main actions for a practical reform towards responsible research assessment.

**Table 1.** The ARRA four core commitments ( Arentoft et al., 2022, pp. 4–6).

Commitment	Purpose
1: Recognise the diversity of contributions to, and careers in, research in accordance with the needs and nature of the research.	This commitment will broaden recognition of the diverse practices, activities and careers in research, considering the specific nature of research disciplines and other research endeavours.
2: Base research assessment primarily on qualitative evaluation for which peer review is central, supported by responsible use of quantitative indicators.	This commitment will enable the move towards research assessment criteria that focus primarily on quality, while recognising that responsible use of quantitative indicators can support assessment where meaningful and relevant, which is context dependent.
3: Abandon inappropriate uses in research assessment of journal- and publication-based metrics, in particular, inappropriate uses of Journal Impact Factor (JIF) and h-index.	This commitment will reduce the dominance of a narrow set of quantitative journal- and publication-based metrics.
4: Avoid the use of rankings of research organisations in research assessment.	This commitment will help avoid that metrics used by international rankings, which are inappropriate for assessing researchers, trickle down to research and researcher assessment. It will help the research community and research organisations regain the autonomy to shape assessment practices, rather than having to abide by criteria and methodologies set by external commercial companies. This could include retaining control over ranking methodologies and data.

The reform promoted by the Coalition for Advancing Research Assessment (CoARA) was created to encourage qualitative assessment that values and highlights the diversity of research activities and Open Science practices ( Di Donato, 2024), and provides for the application of the principles and commitments contained in the ARRA ( Arentoft et al., 2022, p.3).

CNR is the largest interdisciplinary public research organisation in Italy and consists of 7 Departments and 88 Institutes, located in nearly every region of the country. CNR’s human resources comprise around 9,707 employees ^1^.

CNR’s mission, as defined in its Statute ^2^, is to perform research in its own institutes to promote innovation and competitiveness of the national industrial system, to promote the internationalisation of the national research system, to provide technologies and solutions to emerging public and private needs, to advise Government and other public bodies, and to contribute to the qualification of human resources.

A Reorganisation and Relaunch Plan, released in November 2022, established a Reform of CNR through a 3-year program (2023–2025) and beyond, implemented by adapting internal regulations through a step-by-step Roadmap and a validation process. Reforming internal research assessment is one of the milestones of the Roadmap. In the same period, on 7 November 2022, CNR signed the ARRA and joined CoARA. Six months later, CNR announced four competitive calls for career advancement of staff researchers and technologists, operationalising the principles and commitments of the ARRA as a novel evaluation framework.

The present study analyses career progressions at the individual level, a periodic researcher evaluation process designed and performed internally by CNR. Our research aims to verify the extent of implementation of the ARRA commitments across the four career advancement procedures announced in July 2023 for researchers and technologists ^3^.

Each of the four main calls for applications translates into different (and independent) evaluation procedures based on Career Advancement Fields (CAFs), which correspond to CNR disciplinary clusters. In fact, once CNR has established the guidelines through the Call for Applications and the attachments, the procedure requires that the evaluation committees, divided into CAFs, have considerable autonomy in defining the specific evaluation criteria. For each CAF, a certain number of positions are assigned in proportion to the number of eligible candidates.

The present research, therefore, analyses the criteria established by the evaluation committees to examine the degree of alignment between their criteria and the ARRA’s core commitments, vision, and mission to empower Open Science practices.

The remainder of this paper is structured as follows. Section 2 presents an overview of studies on the implementation of CoARA commitments that primarily focus on the transition from high-level commitments to the formulation of institutional policies. Section 3 focuses on the specific context of CNR regulations for career advancement of researchers and technologists, while Section 4 presents an overview of collected data and illustrates the adopted methodology. Section 5 shows the results of the analysis of the adoption of the core commitments in the procedures for career advancement. Section 6 is a discussion of the results shown in the analysis and presents some considerations on possible developments of this work.

## 2. State of the art in implementing the ARRA

The reform of research assessment has become an increasingly prominent issue across institutional and regional contexts, driven by initiatives such as DORA ( San Francisco Declaration on Research Assessment, 2012), the Leiden manifesto ( Hicks et al., 2015), CoARA and the ARRA ( Arentoft et al., 2022).

The ARRA has garnered substantial international support, with 923 signatories. ^4^ As institutions are in the midst of revising their policies to align with responsible research assessment (RRA) principles, recent studies have sought to analyse assessment policies and frameworks. These investigations rely on large-scale surveys ( CoARA WG ACA, 2024a; Hyrkkänen et al., 2023; UK Reproducibility Network et al., 2024) and statistical models ( Lim et al., 2025) to identify trends and attitudes towards assessment reform, as well as institutional policy shifts.

The CoARA working group (WG) Reforming Academic Career Assessment (ACA) carried out a survey in 2024 to gather initiatives at the institutional level seeking to broaden the criteria and methods for assessing the outcomes and impacts of academic activities in recruitment, performance assessment and career progression of academic staff ( CoARA WG ACA, 2024a). Survey respondents represented mainly universities (62.8%), even though a significant share (14.5%) of respondents represented other research-performing organisations. Similarly, the EU-funded project GraspOS ^5^ conducted a landscape survey in 2023 to provide a panorama of responsible research assessment practices in 54 organisations (41 universities and 6 research centres) across 19 European countries ( Hyrkkänen et al., 2023). In the same year, the Open and Responsible Researcher Reward and Recognition (OR4) project also surveyed 59 higher education institutions in the UK on research assessment reform and open research policies ( UK Reproducibility Network et al., 2024).

Survey results indicate increasing engagement with evaluation frameworks aligned with the ARRA, with many institutions actively discussing and/or addressing the commitments ( CoARA WG ACA, 2024a). However, recognition of alternative contributions in research assessment is slow, with research, education, and supervision still largely prioritised, while less traditional aspects such as societal impact and science communication remain largely under-evaluated ( CoARA WG ACA, 2024a; Hyrkkänen et al., 2023). A previous study examining research quality indicators in institutional review, promotion, and tenure policies across seven countries ( Pontika et al., 2022), found that criteria related to open and responsible research were rare, with minimal mentions of Open Access or data sharing. In fact, while most organisations view research assessment as a strategic priority, traditional publication-based metrics remain dominant ( Hyrkkänen et al., 2023). This is reinforced by the findings of CoARA WG ACA, which highlight the gap between discussions on CoARA commitments and their actual implementation. While 70.9% of institutions reported that reform is either underway or planned, only 10.9% indicated full implementation of changes ( CoARA WG ACA, 2024b).

Interestingly, the OR4 survey found that while 75% of surveyed institutions are undertaking strategic efforts to reform research assessment, a significant misalignment persists between RRA and promotion policies. Inconsistencies are reported in approximately 53% of institutions that have both an RRA and a promotion policy, with some emphasising JIF and citation counts, in direct contradiction with RRA principles ( UK Reproducibility Network et al., 2024). The persistence of problematic practices, such as relying on the JIF as a proxy for measuring quality, was also noted ( Pontika et al., 2022).

While these studies offer insights into the evolution of institutional research assessment frameworks in the context of CoARA, they primarily focus on the transition from high-level commitments, such as the signing of the ARRA, to the formulation of institutional policies. As such, they tend to overlook the next critical step: implementing these policies in practice. As a matter of fact, the literature remains limited in addressing the alignment between stated policy and actual practice.

This study seeks to fill that gap by examining whether there are discrepancies between institutional policy and its implementation in the reform of research assessment at CNR. To the best of our knowledge, this is the first study to conduct a detailed analysis of the actual implementation of ARRA Commitments in career advancement calls at the institutional level of a national research performing organisation in relation to its strategic orientations and formal policies.

To this end, our study proposes its own framework for interpreting the alignment with ARRA and CoARA principles, contributing to the ongoing debate on their practical interpretation and implementation. For example, while some members of the scientometric community perceive the ARRA as a tout-court rejection of the use of metrics and indicators ( Abramo, 2024; Torres-Salinas et al., 2023), others align more closely with the underlying spirit of the reform ( Rushforth, 2023) and the position of the CoARA Steering Board, which advocates for a balanced integration of qualitative peer review and quantitative metrics ( Balboa et al., 2024).

## 3. Research evaluation at CNR

The CNR has legal personality under public law as established by Article 2 of Legislative Decree No. 127 of 4 June 2003, and enjoys scientific, financial, organisational, patrimonial and accounting autonomy. It operates under the supervision of the Ministry of University and Research. As a national research entity, it has general scientific competence and maintains scientific institutes distributed throughout the territory.

The fundamental regulations regarding researcher evaluation, and more specifically, recruitment and career advancement, are:

a) the Italian Constitution, which states that the recruitment of personnel must take place by public competition ^6^;
b) dedicated laws, as the Decree-Law DL 127/2003, which (art 20, reorganisation of the CNR) establishes that the presidents of the committees must be full professors or Research Directors, and introduces the rule of the external (independent members belonging to external organisations) prevalence in the assessment committees;
c) The national contract CCNL (area ricerca) – CCNI CNR. Specifically, career advancement for researchers and technologists is governed by Article 15, paragraph 5, of the National Collective Labour Agreement of 7 April 2006; in the CCNI is established that this kind of competition should happen every two years;
d) the PTA, including a staffing needs plan for the triennium;
e) CNR bylaw (which establishes CNR mission/statuto-regolamenti, calls).

**Figure 1.**
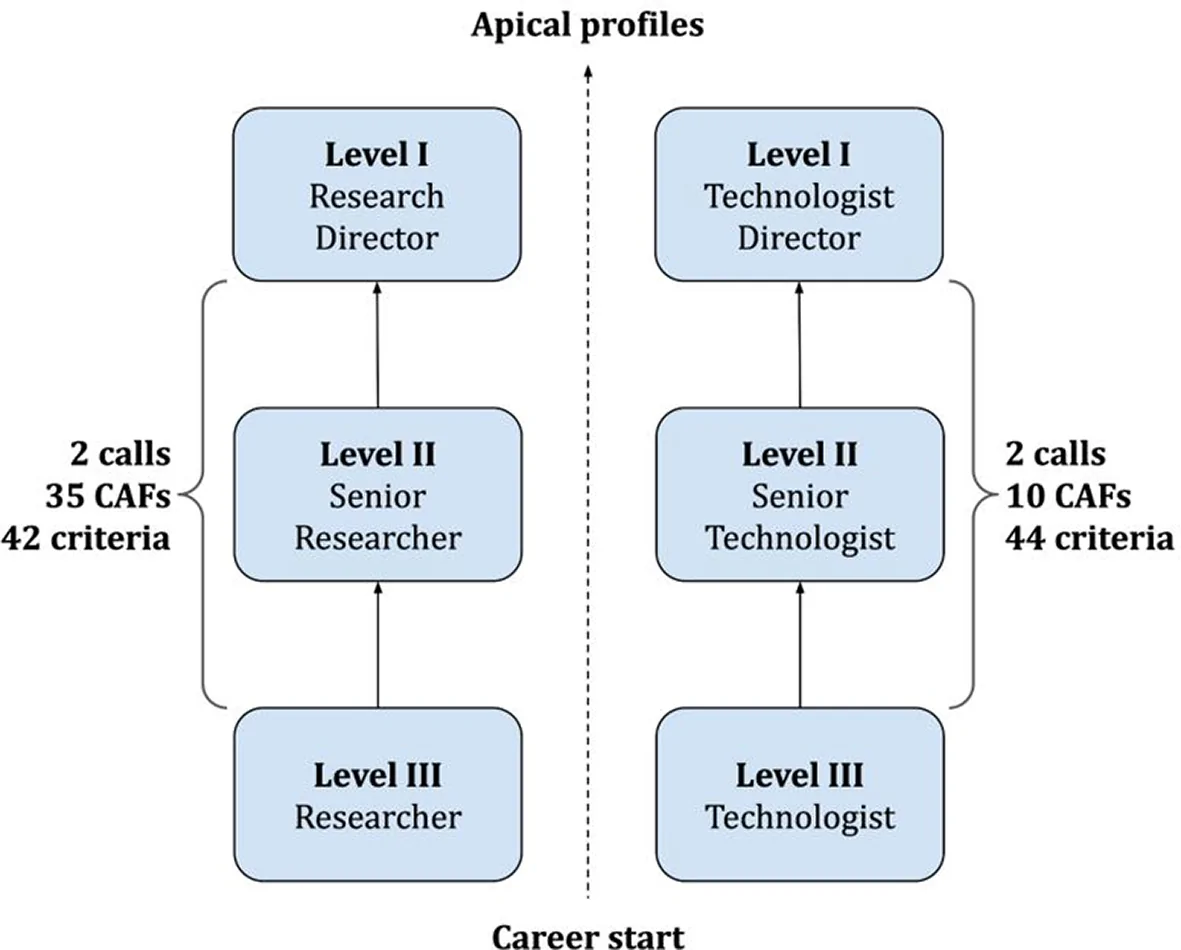
Career profiles for researchers and technologists.

Career levels for researchers and technologists are structured in three tiers for each professional profile (see Figure 1).

In the last competition round, the calls for technologists were divided into 10 CAFs based on the activities carried out and the department of reference, while the calls for researchers were divided into 35 CAFs based on the disciplinary cluster of reference.

The structure of the application in these competitive calls is as follows (see Table 2): candidates had to submit a predefined number of selected products and qualifications (hereafter, for short, P&Qs) in CV Section I. In addition, they were required to write a narrative curriculum, i.e., CV Section II - Contributions and results, which is structured in 3 parts. This section opens with the candidate’s self-assessment across 3 dimensions (researchers) and 10 (technologists) that reflect all aspects of the CNR’s mission. Section II is therefore structured in such a way as to reflect, in the narrative part, the dimensions anticipated in the self-assessment; CV Section III - Perspectives and potentials; CV Section IV - Professional trajectory.

**Table 2.** Subdivision of total points across the different CV Sections and other factors.

	CV						
	P&Qs portfolio	Narrative					
	Sec. I	Sec. II	Sec. III	Sec. IV	Seniority	Oral	Shortlist
**Research Director**	60	30	5	5	—	—	75/100
**Senior Researcher**	45	25	5	5	10	10	60/100
**Technologist Director**	40	25	5	10	—	20	75/100
**Senior Technologist**	30	25	5	10	10	20	60/100

Each call sets out the terms and conditions for participation in the selection process, specifying: the maximum score that can be achieved in the evaluation phase, the allocation of scores in the various sections of the CV, professional experience and interview (where applicable), as well as the minimum score required to be included in the ranking.

In addition to the calls, which explicitly mention the ARRA core commitments and are mainly regulatory in nature, the CNR provided a series of attachments to assist candidates in submitting their applications. These documents, which have regulatory value as annexes to the call for applications, also provided the evaluation committees with a high-level overview of the criteria to be used in evaluating candidates.

Specifically, four attachments were provided for this selection process:

**Attachment A. Professional CV guidelines** ^7^. This document provides general guidelines for compiling a narrative CV and, among other information, the principles underlying the evaluation process, with explicit reference to the CoARA agreement signed by the CNR in 2022, highlighting the main changes compared to previous selection processes. The guidelines describe how the CV should be structured, the information that each CV Section must contain, and the relative scores, broken down by Researchers and Technologists, as shown in the table above.

**Attachment B. Professional CV template**. Four separate templates, one for each call. They include CV Sections II, III and IV mentioned in the calls.

**Attachment C. Selected products and qualifications.** Two templates, one for researchers and one for technologists, containing a comprehensive list of all products and qualifications eligible for evaluation and the relevant forms to be used for submission. The products are grouped by macro-type (e.g., contribution to a journal), and each macro-type specifies subcategories (e.g., the sub-types of contribution to a journal include: article, letter, review, perspective, software/data paper, bibliographic reference, translation, note on a judgement). Each form specifies a list of mandatory and optional metadata and narrative elements (e.g., impact, multidisciplinarity). This part constitutes CV Section I of the application.

**Attachment D. Technical attachment on the products/titles in CV Section I**. This is a single document for all calls that describes the procedure and rules for confirming the products and qualifications to be submitted for evaluation.

**Figure 2.**
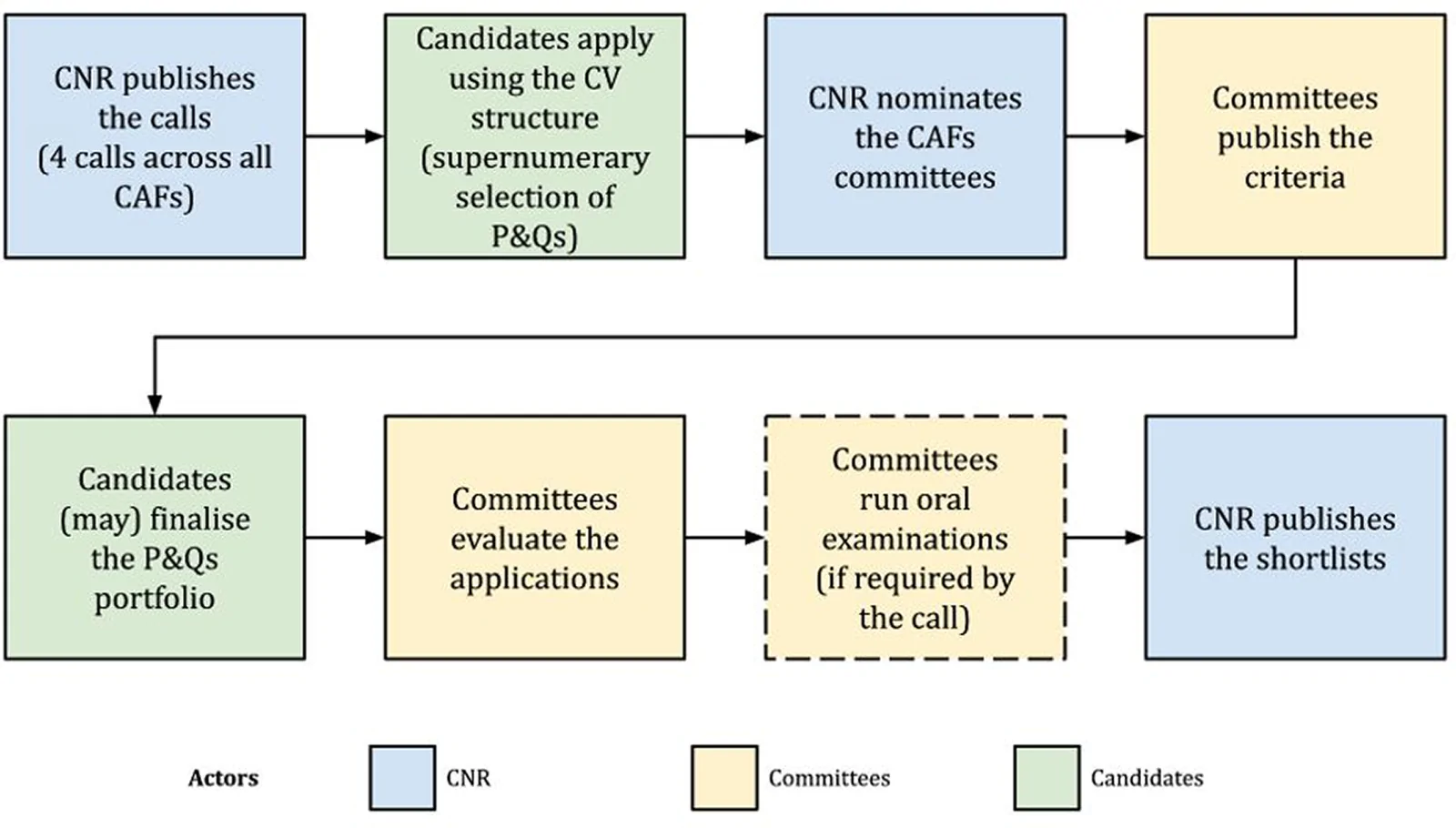
Structure of the application procedure.

Figure 2 shows the assessment process, which is mediated by the CNR’s portal for online application ^8^.

## 4. Data and methods

The calls and the attachments have been collected by accessing the official documents published on the CNR’s public relations office portal.

The documents analysed in this paper are listed in Annex F. They include the ARRA, the official profile descriptions reported in the national collective agreement for researchers and technologists, the four calls of selection and their attachments (A, B, C, D), as well as the evaluation criteria for the 90 committees and related errata corrige and clarifications to the criteria. To facilitate the analysis and reproducibility of the results described in this paper, the PDF files relevant to our study were downloaded via web scraping and archived. The Python code and the collected data are available in a public repository on GitHub ^9^ and on Zenodo ( Mannocci, 2026).

The two calls targeting researchers bring into evaluation 11 different research products and 31 different qualifications for a total of 42 possible items (and relative criteria) for us to analyse. The two calls targeting technologists bring into evaluation 11 different research products and 33 qualifications, for a total of 44 possible items (and relative criteria) for us to analyse. Moreover, the 90 committees established dedicated criteria for sections II-IV (narrative CV). This yields a total of 3,820 criteria to analyse for section I + 270 for sections II-IV ^10^, for a total of 4,090 criteria.

The collected documentation has been analysed to verify the compliance between the criteria adopted by the evaluation committees and CoARA four core commitments and the recognition and valorisation of Open Science practices. In order to do so, the research team developed a matrix based on a set of questions which guided the analysis (see Annex E).

Commitment 1 requires to “Recognise the diversity of contributions to, and careers in, research in accordance with the needs and nature of the research”, with the purpose of broadening “recognition of the diverse practices, activities and careers in research, considering the specific nature of research disciplines and other research endeavours”.

In order to analyse the compliance with Commitment 1, we analysed the effective potential score attributed to each criterion in Section I (for a total of 3,820 criteria). Full compliance is considered achieved only when all research products and qualifications may receive the maximum potential score. We also collected data on the average score, the deviation in scores for product categories and securities (by commission and then by product type/qualification), and the number of products and qualifications that were assigned a default score of zero.

Commitment 2 asks to “Base research assessment primarily on qualitative evaluation for which peer review is central, supported by responsible use of quantitative indicators”, with the purpose to “enable the move towards research assessment criteria that focus primarily on quality, while recognising that responsible use of quantitative indicators can support assessment where meaningful and relevant, which is context dependent”.

Therefore, to determine whether a criterion is primarily qualitative or quantitative, we considered the following guiding questions: Can the research product or qualification be assessed without examining its content? Are the points assigned predominantly (i.e., over 50% of the total score) based on a mechanical or purely metric-based evaluation? If the answer to either question is yes, the criterion is considered to violate the ARRA Commitment #2 and is flagged as predominantly quantitative. Conversely, if a criterion requires the committee to directly review the content of the product or qualification, it is annotated as mostly qualitative and therefore deemed aligned with ARRA Commitment #2. In general, we resolved the most intricate cases via discussions and by adopting an agreed-upon understanding and annotation strategy. However, whenever a criterion was too obscure or raised an unresolvable perplexity, we opted to annotate it as partially fitting. We applied the research questions to the 3,820 Section I criteria and to the 270 Section II-IV criteria, for a total of 4,090 criteria.

In general, a criterion was deemed not qualitative if it was applied mechanically, overly rigid, or purely numerical (e.g., 0.1 points for every 10 hours of teaching). Similarly, a criterion was considered not qualitative if no specific guideline was provided, leading to an automatic score assignment. Internationalisation was deemed not qualitative when applied mechanically (e.g., systematically assigning higher scores to products published in international outlets) or when it was irrelevant to the profile ^11^. Finally, authorship role was considered not qualitative if it referred merely to a formal role (e.g., first author, co-author) and was applied mechanically, without regard for the content and quality of the author’s actual contribution. We are aware that there are disciplinary fields in which it is not customary to define each person’s contribution to the final work, sectors where it is clear that the position indicates each person’s contribution to the work (first name, middle name, last name) and sectors where each person’s contributions are declared. However, the division into macro-disciplinary areas carried out by the CNR with the choice of CAFs does not always allow for the identification of a single area per CAF (for example, in CAF35 of the calls for researchers, “Philosophical, linguistic, philological-literary sciences and their digital applications” covers several disciplinary areas including Italian studies, philology, linguistics, philosophy and digital humanities - which also include careers in ICT sectors). Moreover, the explicit reference to multidisciplinarity as something to be valued renders this type of criterion inapplicable in the calls in question. Thus, the mechanical application of a numerical factor without considering the actual quality of the contribution was considered a quantitative factor.

Commitment 3 requires to “Abandon inappropriate uses in research assessment of journal- and publication-based metrics, in particular inappropriate uses of Journal Impact Factor (JIF) and H-index” and defines inappropriate uses as “relying exclusively on author-based metrics (e.g., counting papers, patents, citations, grants, etc.) to assess quality and/or impact; assessing research products based on metrics relating to publication venue, format or language; and relying on any other metrics that do not properly capture quality and/or impact.

We thus assessed the use of publication- and journal-based metrics in assigning evaluation scores, considering an infringement of Commitment 3 where research products were evaluated based on the venue (e.g., research products from certain outlets were considered inherently superior), language (e.g., English-language products were systematically favoured), or format (e.g., specific product subcategories were intrinsically valued more than others). Given the focus of the commitment, criteria on Qualifications and on sections II-IV (narrative CV) were not considered in this analysis.

Finally, ARRA Commitment 4 asks to “Avoid the use of rankings of research organisations in research assessment” with the purpose to “help avoid that metrics used by international rankings, which are inappropriate for assessing researchers, trickle down to research and researcher assessment”.

Therefore, we considered whether rankings of any kind (e.g., university rankings) are used to assess the quality of research products or qualifications, and if they affect the evaluation of Section I of researchers’ CVs.

As the ARRA recognises Open Science practices essential both for the Principles for overarching conditions and the Principles for assessment criteria and processes ( Arentoft et al., 2022, pp. 3–4), on top of verifying the compliance towards the four ARRA commitments, we verified if and when Open Science and related practices (i.e., open access, open source, FAIR open data, early sharing practices, open methods and processes, open peer review, and openness to the lay public, such as public engagement and citizen science) were explicitly mentioned in the evaluation criteria of Sections I-IV.

All members of the research group first analysed the criteria individually. At the end of this initial analysis, from February to July 2025, fifteen collegial meetings were carried out by the four authors of this paper to confront and discuss any disagreements. The results of our analysis have been thoroughly reported in structured spreadsheets, which are openly available on Zenodo as underlying data ( Di Donato et al., 2026a) ^12^.

## 5. Analysis

This section presents the aggregated results of our analysis, following the methodology outlined in the previous section.

### 5.1 Commitment 1: Recognise the diversity of contributions to, and careers in, research in accordance with the needs and nature of the research

In this section, the analysis of the compliance between ARRA Commitment 1 and the maximum potential scores assigned by the evaluation committees to products and qualifications (Section I) is summarised. Table 3 presents the analysis related to the calls for researchers (Research Directors and Senior Researchers).

**Table 3.** Analysis of evaluation criteria used by the committees against Commitment 1 (Researcher Directors, Senior Researchers).

Call for Research Directors	Call for Senior Researchers
**Analysis by CAF (see** Figure 3 **)** 5,7% of CAFs are fully compliant, assigning the maximum score of 3 to all products and qualifications alike. In the remaining 94.3%, less than 50% of products and qualifications are eligible for the maximum score (the highest proportion being 47.6%). 48.6% assign a 0 score by default to at least one contribution type, effectively excluding certain products and qualifications from the assessment. In the worst case, 33% of contributions are considered to have no value. 60% limit more than 50% of contributions to a maximum score between 0 and 2. In 48.6%, the average score of contributions is at least 1.5, while it exceeds 2 in only 14.3%. **Analysis by P&Q (see** Figure 4 **)** Only one qualification (institute director, representing 2.4% of the total) is consistently eligible for the maximum score of 3. In addition, 7.1% of products and qualifications (journal contributions, project coordination, and ERC grant) receive the maximum score from 95% of CAFs. In contrast, 45.2% of contributions receive a default score of 0 in at least one CAF. For these contributions, potential scores range from 0 to 3, reflecting significant variations in how different CAFs value them. **Average score (products)**: 1.6 **Average score (qualifications)**: 1.6 **Average score (products + qualifications)**: 1.6 **Standard deviation**: Contributions with a high mean score (close to 3 points) tend to have a low standard deviation (σ < 0.4), indicating that their importance is widely recognised across committees. Conversely, contributions with lower average scores tend to exhibit higher standard deviations, reflecting greater uncertainty regarding their value, and they should be evaluated.	**Analysis by CAF (see** Figure 5 **)** 0% of CAFs are fully compliant, meaning that no committee assigns the maximum score of 3 to all products and qualifications. 34.3% explicitly exclude at least one contribution type from the assessment. 17.1% apply a 0 score by default to more than 20% of contributions (with two cases accounting for 33.3% of the total products and qualifications). 45.7% limit more than 50% of contributions to a maximum score between 0 and 2. In 60%, the average score of contributions is at least 1.5, while it exceeds 2 in only 14.3%. **Analysis by P&Q (see** Figure 6 **)** Across all CAFs, less than 45% of products and qualifications are eligible for the maximum score of 3. Only two qualifications (project coordination and institute director, representing 4.8% of the total) are consistently assigned the maximum score. Journal contributions have an average maximum score of 2.98; however, in several CAFs, subtypes of journal contributions are systematically disadvantaged, with only the subtype “Journal article” consistently eligible for a score of 3. 42.9% of contribution types are deemed ineligible for assessment and are assigned a default score of 0 in 34.2% of committees. Overall, both product and qualification categories follow a descending pattern of maximum scores. It seems that the numerical order of the contributions listed in Attachment C of the call was interpreted as a ranking in terms of value. This trend is observable for both products and qualifications: for example, products P1 to P6 are generally assigned higher potential scores, while P7 to P11 receive lower maximum scores. A similar pattern is visible in the criteria set for the qualifications. 40% of products/qualifications have an average maximum score between 0 and 1.50. **Average score (products)**: 1.72 **Average score (qualifications)**: 1.61 **Average score (products + qualifications)**: 1.64 **Standard deviation**: Entire categories of qualifications that are not discipline-specific (such as national scientific qualifications, participation in national or international technical-scientific bodies, and university teaching assignments) are assigned scores ranging from 0 to 3, with a high standard deviation between 0.6 and 1.1.

**Figure 3.**
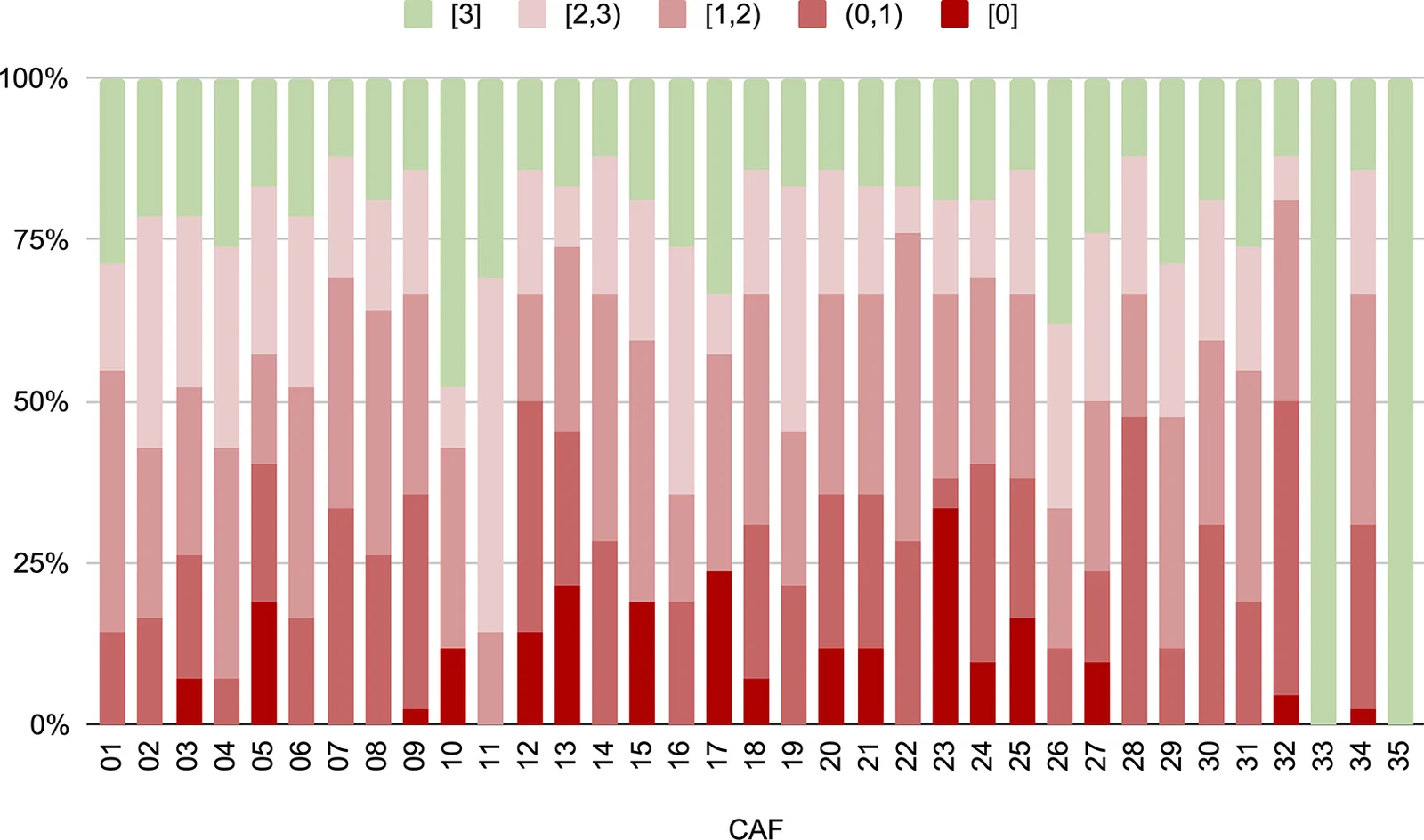
Compliance with Commitment 1 by CAF - Research Directors.

**Figure 4.**
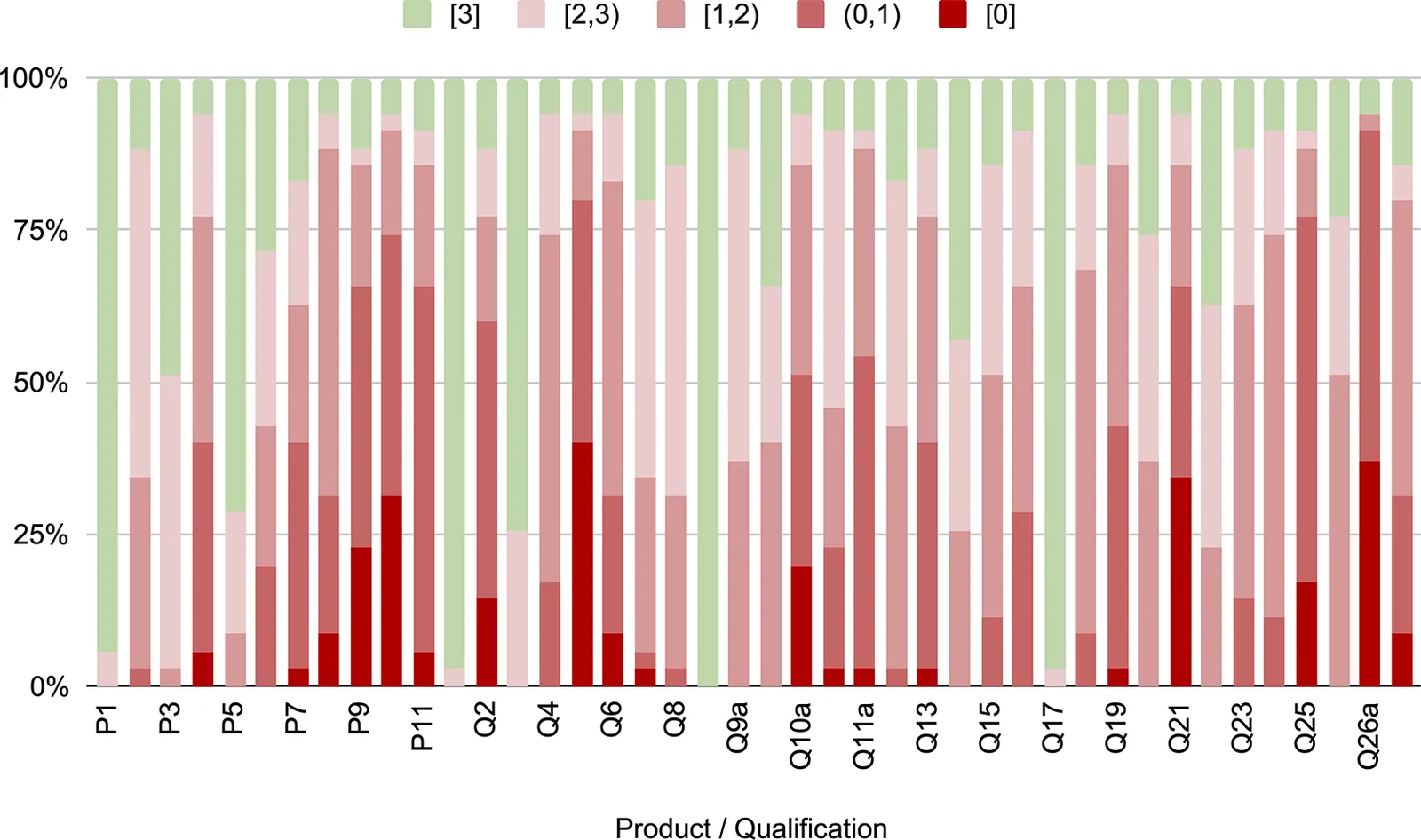
Compliance with Commitment 1 by Product/Qualification - Research Directors.

**Figure 5.**
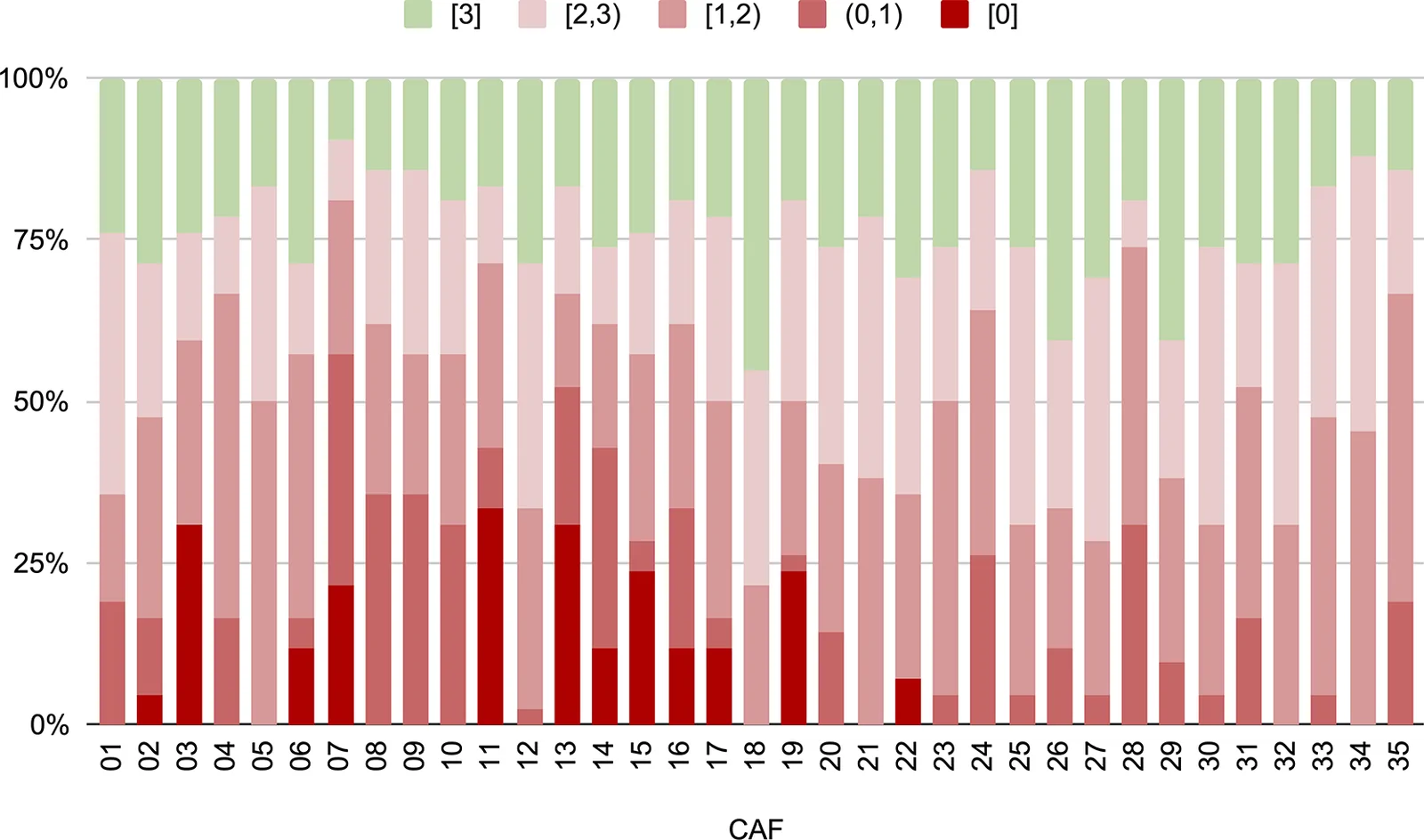
Compliance with Commitment 1 by CAF - Senior Researchers.

**Figure 6.**
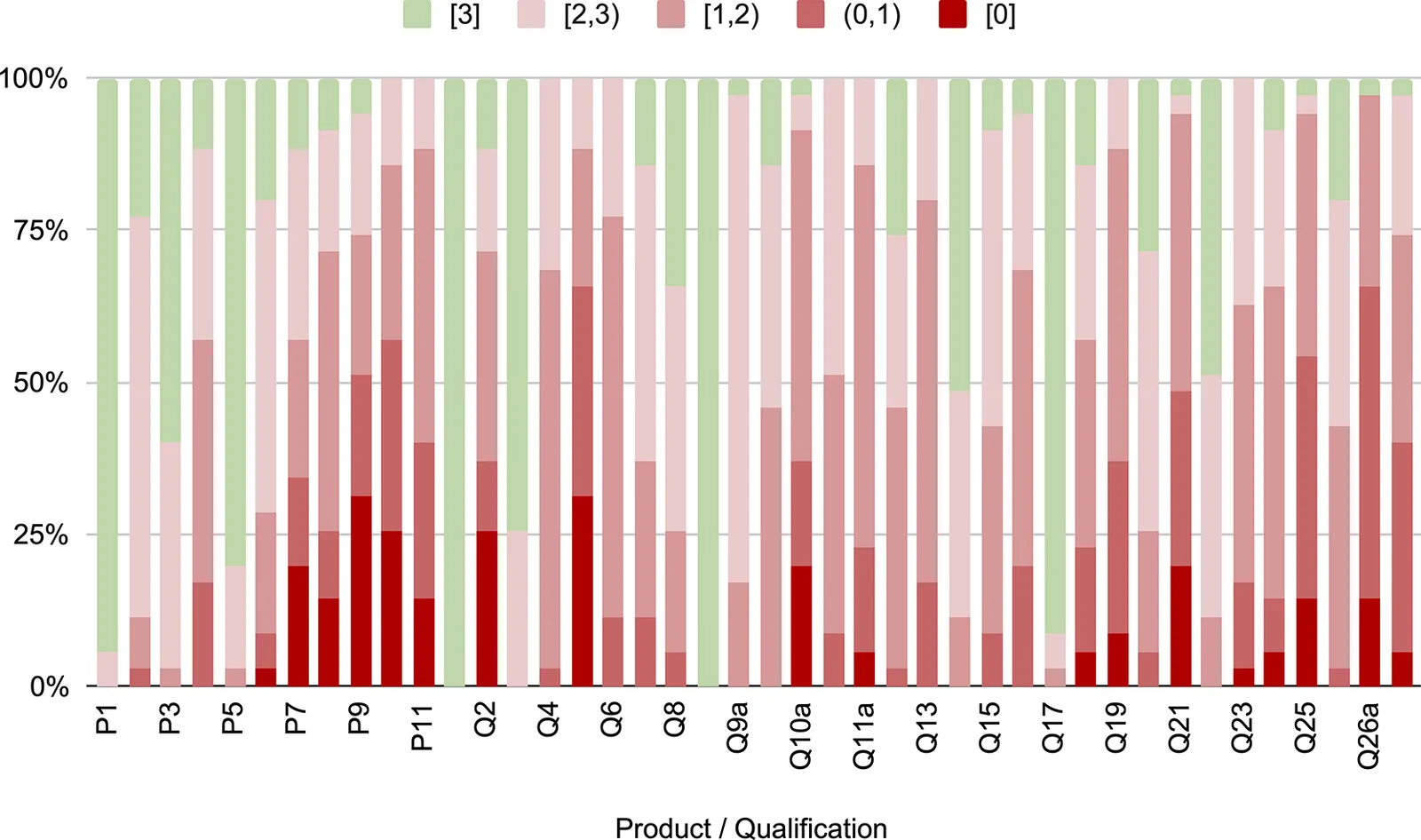
Compliance with Commitment 1 by Product/Qualification - Senior Researchers.

Figures 3 to 6 illustrate the level of compliance with Commitment 1 in the calls for Research Directors and Senior Researchers. To support the analysis of results for Commitment 1 for the CV Section I, we calculated the average score and standard deviation of each product and qualification across CAFs, as well as the average score and standard deviation assigned in each CAF to products and qualifications. Similarly, we aggregated scores by binning them, i.e., [0], (0,1), [1,2), [2,3), [3] for the two calls targeting researchers, and [0], (1,2), (0,1), [1,2), [2] for the two calls targeting technologists.

As can be seen from the figure, only 2 CAFs (both in SSH sectors, i.e., “Historical sciences for the study of cultures and civilisations in a global comparative perspective” and “Philosophical, linguistic, philological-literary sciences and their digital applications”) are fully compliant with Commitment 1.

Only one qualification, Institute Director, has the maximum score across the board. Conversely, entire categories of products and qualifications, accounting for over 51% of the total, have very low average scores, amounting to less than half of the maximum potential score (1.5). This is particularly true for products that fall outside the standard or “main” types (i.e., journal contributions, contributions in volumes and books), and include contributions in conference proceedings and posters, projects, exhibitions, cartographic products, concordances, and technical reports.

The same applies to qualifications, where participation in activities (e.g., projects, research infrastructures, editorial boards, and scientific committees) is undervalued compared to the corresponding roles of responsibility by default. The same applies to the supervision of doctoral students, outreach activities and certain leadership positions.

As shown in the figure, no CAF is fully compliant with Commitment 1.

As observed for Research Directors, the data show that over 40% of products and qualifications have an average maximum score between 0 and 1.50. In particular, this is once again the case for the same product categories considered a priori less important in calls for Research Directors, namely projects and prototypes, exhibitions, cartographic products, concordances, and technical reports.

The same applies to certain qualifications, which, as in the case of Research Directors, undervalue participation activities compared to the corresponding roles of responsibility. Evaluation activities are scored an average of 1.3 points, and science for policy contributions, such as technical-scientific consulting, public engagement activities, technical-managerial assignments within the institution, organisational positions with high professional content, professional qualification and supervision of doctoral students, all have an average score below 1.5.

Table 4 presents the analysis of compliance between ARRA Commitment 1 and the maximum potential scores assigned by the evaluation committees to products and qualifications (Section I) in the calls for technologists (Technologist Directors and Senior Technologists).

**Table 4.** Analysis of evaluation criteria used by the committees against Commitment 1 (Technologist Directors and Senior Technologists).

Call for Technologist Directors	Call for Senior Technologists
**Analysis by CAF (see** Figure 7 **)** 10% are fully compliant with commitment 1. Another 10% are compliant with commitment 1, except for 1 product/qualification. In 40% the scoring limits certain contribution types to scores between 0 and 1, suggesting a notably low level of recognition. In 20% certain types of contributions are excluded from the assessment by default. **Analysis by P&Q (see** Figure 8 **)** 31.8% of product/qualification categories are consistently eligible for the highest score of 2. 55.5% of product/qualification categories have an average maximum score above 1.8, indicating that they are eligible for the highest score in nearly all CAFs, and reflecting a relatively consistent level of recognition across these. 22.7% rarely receive the maximum score and have a mean score below 1.5. **Products average score**: 1.87 **Qualifications average score**: 1.67 **P + Q average score**: 1.72 **Standard deviation**: In general, contributions with high average scores (μ > 1.8) tend to exhibit lower standard deviations (σ < 0.4), indicating a high level of agreement among CAFs regarding the importance attributed to a given product or qualification. On the contrary, contributions with low average scores show a higher standard deviation, reflecting that the value of the type of contribution is not agreed upon across CAFs. This is particularly noticeable for contributions in conference proceedings (μ: 1.36, σ: 0.48), participation in archaeological campaigns (μ: 1.23, σ: 0.48), participation in research infrastructures (μ: 1.28, σ: 0.48), national scientific qualifications (μ: 1.20, σ: 0.80), and professional qualifications (μ: 1.15, σ: 0.63).	**Analysis by CAF (see** Figure 9 **)** 20% are fully compliant with commitment 1. 10% are compliant with commitment 1, except for 1 product or qualification. 0% excluded certain types of contributions by default. **Analysis by P&Q (see** Figure 10 **)** Alignment with Commitment 1 is significantly higher in the evaluation of research products: in 27.3% of CAFs, all products are eligible for the maximum score of 2, while in the other 72.7% the maximum score is assigned to at least 50% of products. Only 21.2% of qualifications receive the maximum score of 2 across all CAFs. 15% of products and 30% of qualifications receive scores between 1 and 2. **Products average score**: 1.75 **Qualifications average score**: 1.65 **P + Q average score**: 1.68 **Standard deviation**: Overall, the standard deviation across all CAFs and contributions is 0.5, with no meaningful difference between products (0.48) and qualifications (0.50).

**Figure 7.**
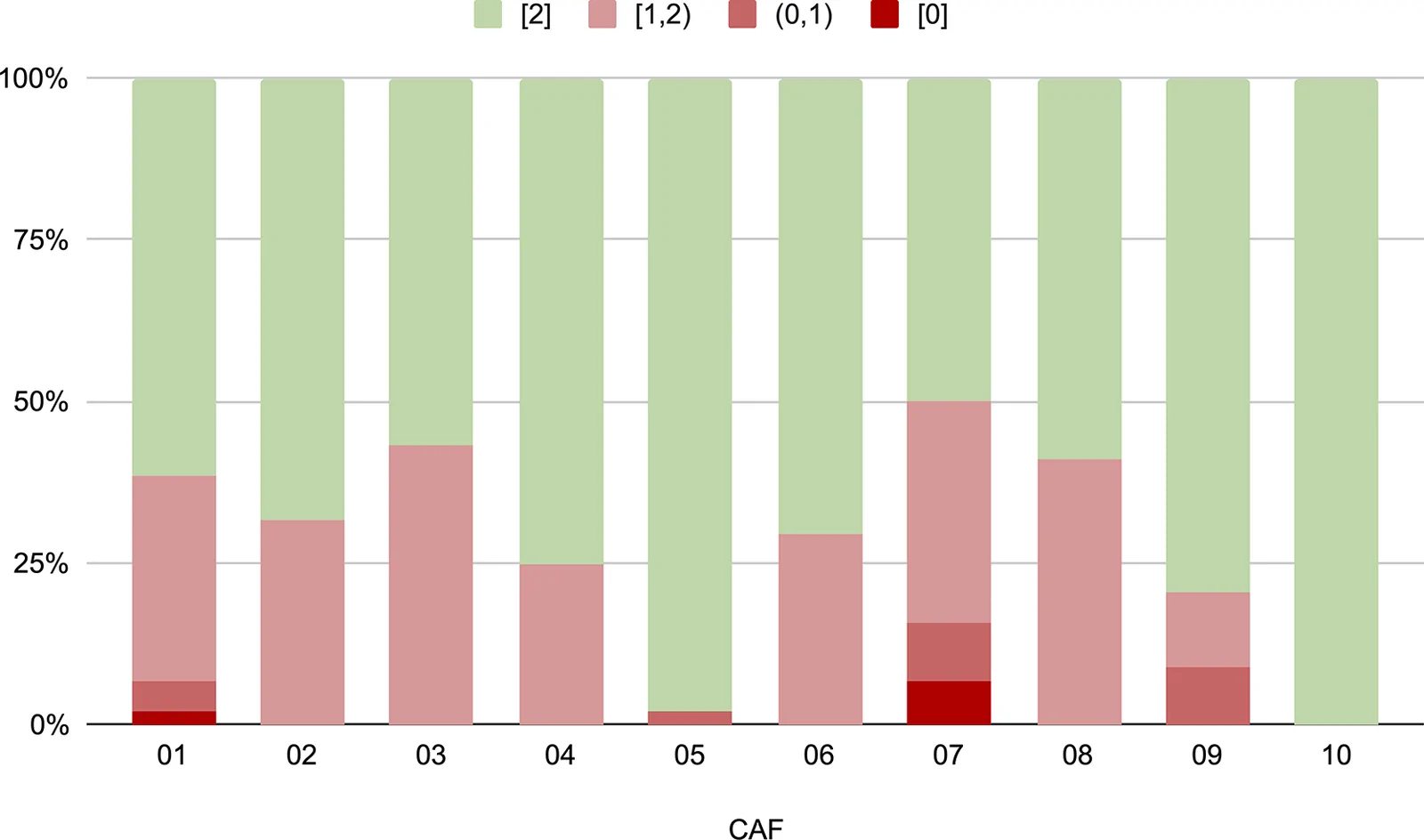
Compliance with Commitment 1 by CAF - Technologist Directors.

**Figure 8.**
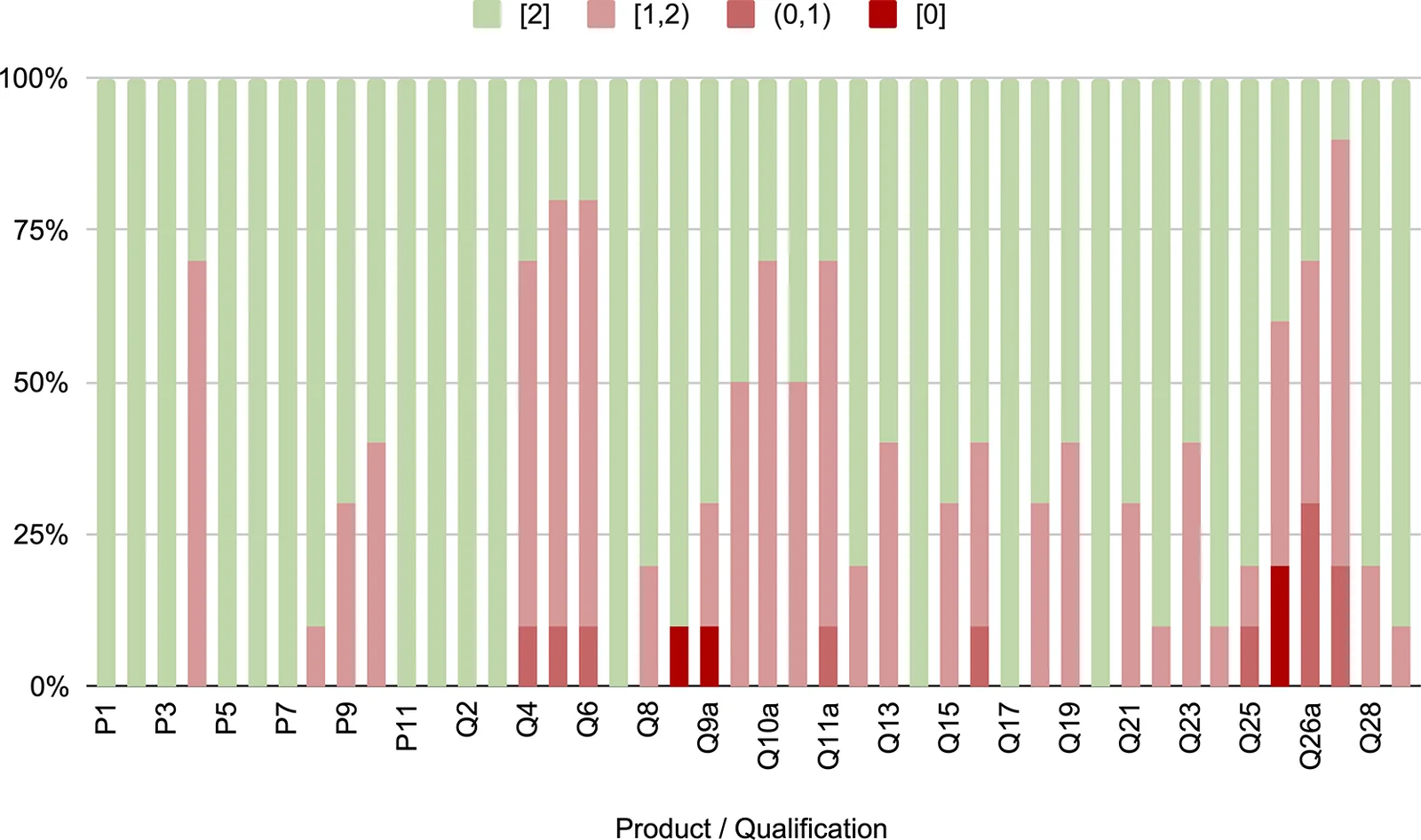
Compliance with Commitment 1 by Product/Qualification - Technologist Directors.

**Figure 9.**
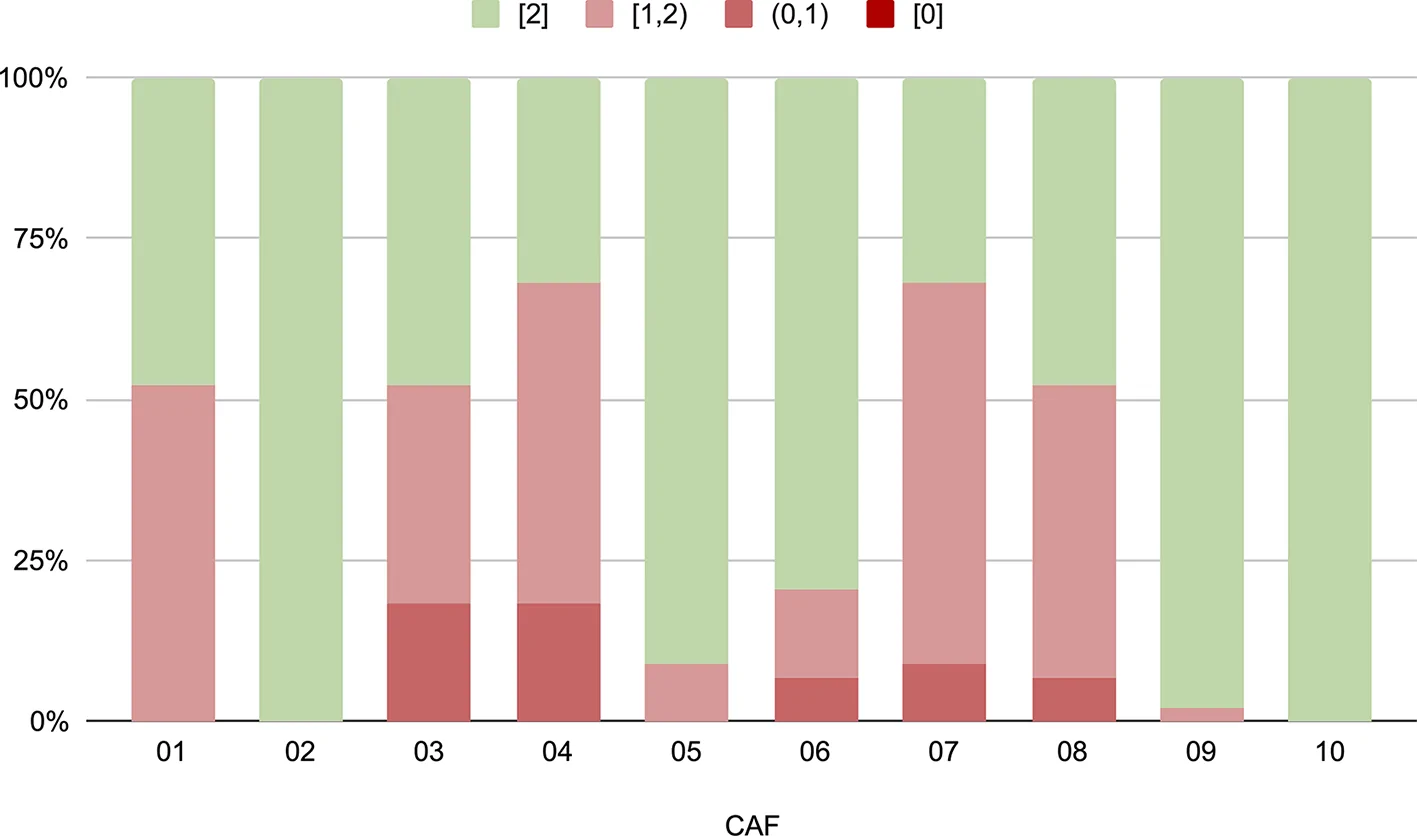
Compliance with Commitment 1 by CAF - Senior Technologists.

**Figure 10.**
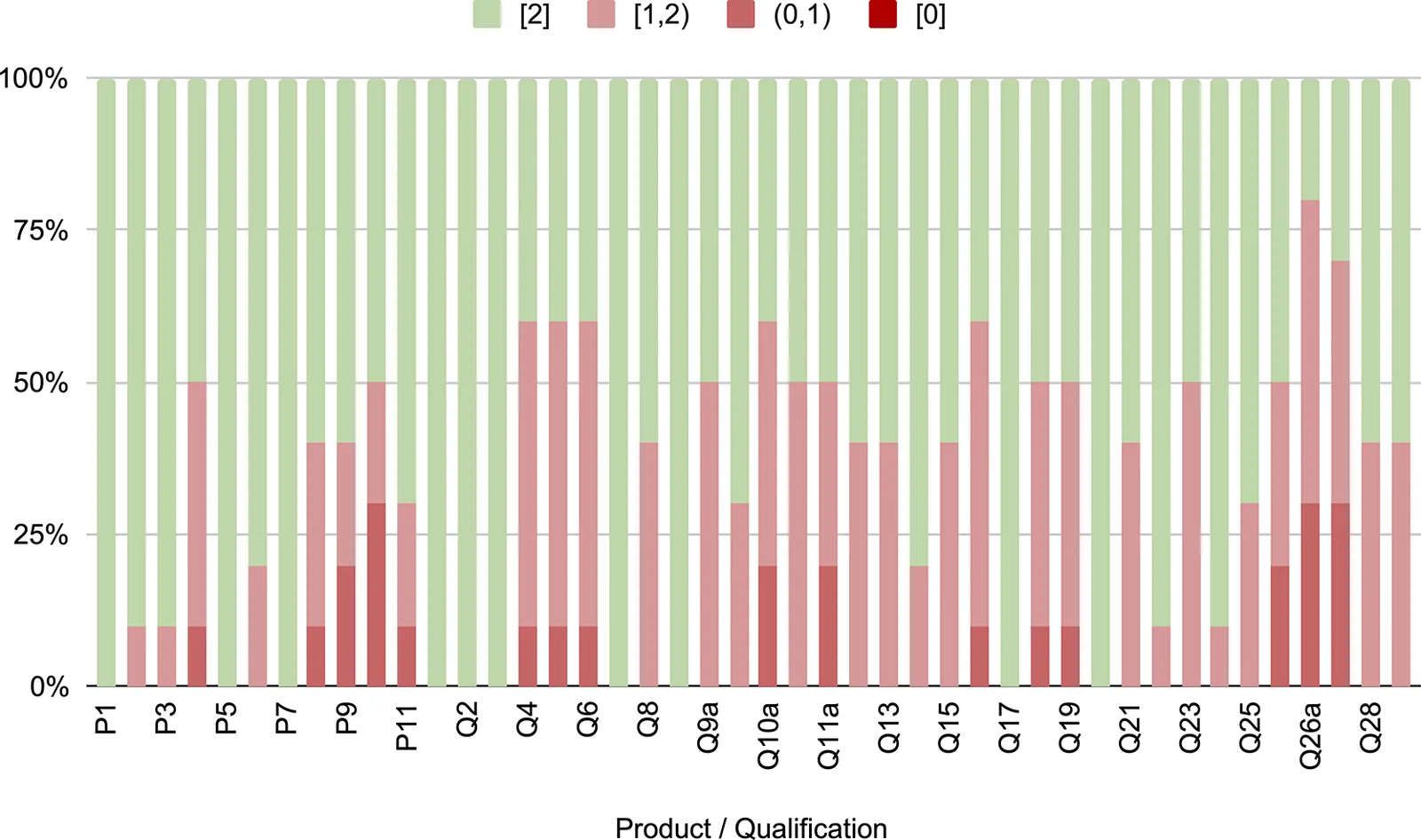
Compliance with Commitment 1 by Product/Qualification - Senior Technologists.

Figures 7 to 10 illustrate compliance with Commitment 1 in the calls for Technologist Directors and Senior Technologists.

Of the 10 CAFs, only one, CAF10 (Decision-making and management processes of the institution), is fully compliant with Commitment 1.

Almost one-third of the products and qualifications receive the maximum score, including 7 out of 11 products (journal contributions, contributions in volumes, books, patents, databases and software, projects, and technical reports) and 7 out of 29 qualifications. Once again, roles related to the coordination or direction of projects, archaeological campaigns, research infrastructures, scientific journals, and technical/scientific studies are valued more highly than participation in the same activities. In the case of Technologist Directors, the awarding of ERC grants or major prizes also receives the maximum score, together with scientific diplomacy assignments.

Other products and qualifications with an average score above 1.8 include exhibitions, fellowships, leadership positions (e.g., director of an institute or scientific society), technical responsibilities, and participation in start-ups and spin-offs.

Among the products, contributions to conference proceedings do not exceed an average score of 1.5, and this also applies to participation in projects, archaeological campaigns, and research infrastructures. Teaching activities, including doctoral student supervision and national scientific qualification, are generally undervalued, as are research evaluation activities.

The highest score is assigned to all products and qualifications in only two CAFs: CAF02 (Bio-agri-food sciences) and CAF10 (Decision-making and management processes of the institution). In the remaining 8 CAFs, different scores are assigned based on the type of contribution.

The analysis reveals a tendency to slightly prioritise research products over qualifications. This difference is mainly explained by the assignment of the maximum score (i.e., 2) to 77% of the 11 research products, compared to 64% of the 33 qualifications. Conversely, 15% of products and 30% of qualifications receive scores between 1 and 2. The remaining contributions are assigned a score between 0 and 1, accounting for 7% of products and 5% of qualifications. Products which are assigned the maximum score across all CAFs include journal contributions, patents, and projects. Product categories such as contributions in conference proceedings and concordances are significantly penalised, receiving the maximum score in only 50% of CAFs.

Among the qualifications, those consistently assigned the maximum score of 2 across all CAFs include coordination roles, institute director positions, ERC grants, and scientific diplomacy assignments. Specifically, leadership roles are often awarded the maximum score of 2, whereas participation and supervisory activities, such as doctoral student supervision, typically receive lower scores. Other highly rated qualifications include participation in start-ups and spin-offs; technical-administrative roles within the institution (assigned high scores in 9 CAFs); direction of scientific journals (in 8 CAFs); and university teaching assignments (in 7 CAFs). By contrast, the most penalised qualifications are professional qualifications (with a maximum score in only 3 CAFs) and doctoral student supervision (with a maximum score in only 2 CAFs).

It is important to underline that in the Senior Technologists calls, no products or qualifications are excluded from the assessment. However, limited scope is given to recognising the diversity of contributions within a technologist’s career, as research products and qualifications are not assessed on an equal basis in most CAFs.

Overall, scoring is generally consistent for journal articles, book chapters, and coordination roles in projects, archaeological campaigns, or research infrastructures. However, discrepancies remain for certain products, such as conference proceedings and cartographic outputs, as well as for some qualifications, including participation in research infrastructures and other teaching assignments. These inconsistencies may reflect the diversity of disciplinary areas represented in the call, which appears to influence how the value of certain contributions is interpreted. For example, contributions in conference proceedings receive 0.5 points in the Humanities but 2 points in ICT. Such divergence stands in contrast with Attachment B of the call, which clearly states that the CVs should be assessed independently of the disciplinary area of the call in order to recognise and value multidisciplinarity.

### 5.2 Commitment 2: Base research assessment primarily on qualitative evaluation for which peer review is central, supported by responsible use of quantitative indicators

In this section, the analysis of compliance with ARRA Commitment 2 and the criteria adopted by the evaluation committees for Section I (selected products and qualifications) and Sections II-IV (narrative CV) is summarised. As explained above, a criterion is considered qualitative and is deemed aligned with ARRA Commitment 2 if it requires the committee to directly review the content of the product, qualification, or narrative CV.

Table 5 below presents the analysis related to the calls for researchers (Research Directors and Senior Researchers).

**Table 5.** Analysis of evaluation criteria used by the committees against Commitment 2 (Research Directors, Senior Researchers).

Call for Research Directors	Call for Senior Researchers
** Section I - Analysis by CAF (** Figure 11 **)** In 57.1% of CAFs, more than 50% of the contributions are evaluated using qualitative criteria. Conversely, in 42.9%, the criteria are not aligned with the commitment due to a predominant reliance on quantitative indicators in the evaluation of at least 50% of P&Qs. Partially fitting criteria are identified at least once in 34.3% of CAFs. ** Section I - Analysis by P&Q (** Figure 12 **)** When analysing the criteria used to assess contribution types across CAFs, 69% are evaluated using mostly qualitative criteria. Conversely, 16.7% of contribution types are evaluated primarily using quantitative criteria. In general, products are evaluated primarily using qualitative criteria; the only exceptions are concordances and technical reports, where qualitative and quantitative criteria are almost evenly balanced. Partially fitting criteria are applied at least once in the evaluation of 19% of contributions. **Sections II-IV** The criteria set out to evaluate the Narrative CV sections are largely compliant with CoARA Commitment 2, with a few notable exceptions. In particular, unlike other CAFs, 1 CAF does not provide detailed criteria for CV Sections II, III, and IV, including only criteria for research products and qualifications. Additionally, 1 CAF lacks detailed criteria for CV Sections II and IV, making it unclear how scores are assigned, despite the evaluation being described as collegial.	** Section I - Analysis by CAF (** Figure 13 **)** In 45.7% of CAFs, more than 50% of the contributions are evaluated using qualitative criteria. In 20%, qualitative assessment is applied to more than 95% of the contributions. Conversely, in 54.3% of cases, the criteria are not aligned with the commitment due to a predominant reliance on quantitative indicators in the evaluation of at least 50% of contributions. In some cases, evaluation criteria for certain contributions are entirely absent. In 65.7%, partially fitting criteria are identified at least once. In two CAFs, at least half of the criteria established by the committee are deemed partially fitting. ** Section I - Analysis by P&Q (** Figure 14 **)** Most products are evaluated primarily using qualitative criteria. Only patents are associated with a relative majority of quantitative criteria. In contrast, 29% of qualifications are associated with an absolute majority of quantitative criteria or with no qualitative criteria. 11.4% reflects an equal distribution of qualitative and quantitative criteria. Partially fitting criteria are applied at least once in the evaluation of 81% of contributions. **Sections II-IV** The criteria established to evaluate the Narrative CV sections are compliant with Commitment 2. In 5.7% of CAFs, the criteria set out for Section II are partially fitting, and in 2.8% the criteria for Section III are partially fitting.

**Figure 11.**
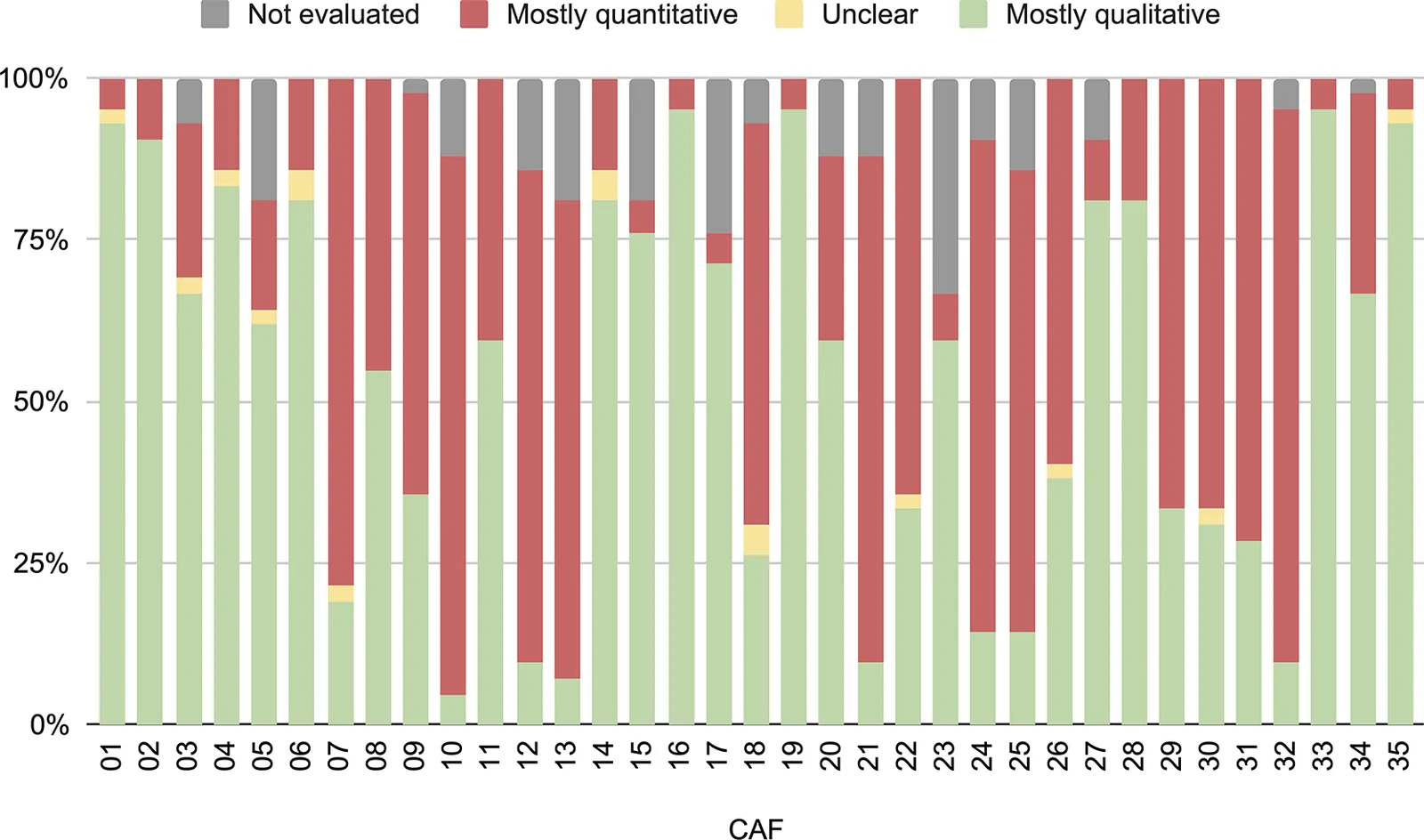
Compliance with Commitment 2 by CAF (Section I) - Research Directors.

**Figure 12.**
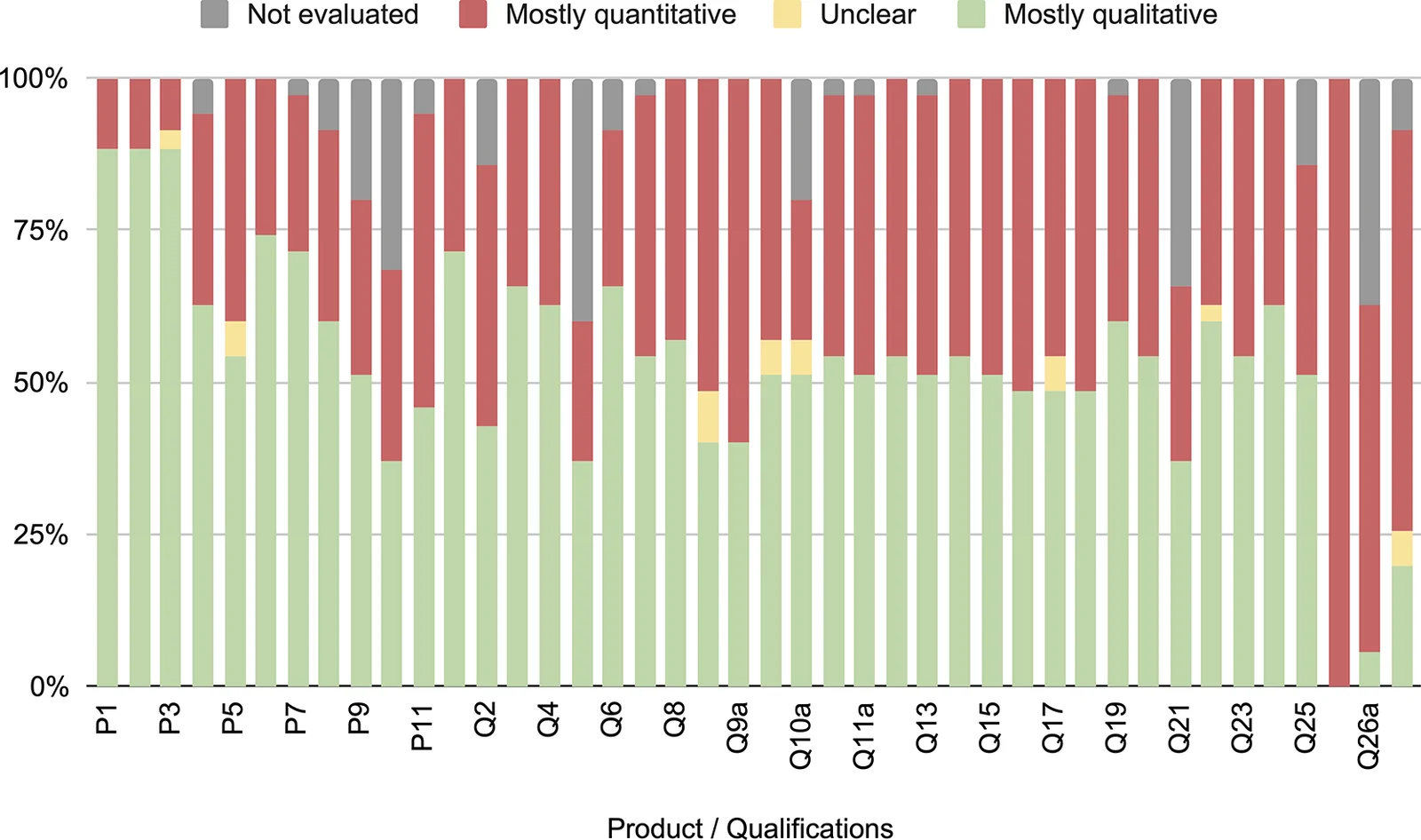
Compliance with Commitment 2 by Product/Qualification (Section I) - Research Directors.

**Figure 13.**
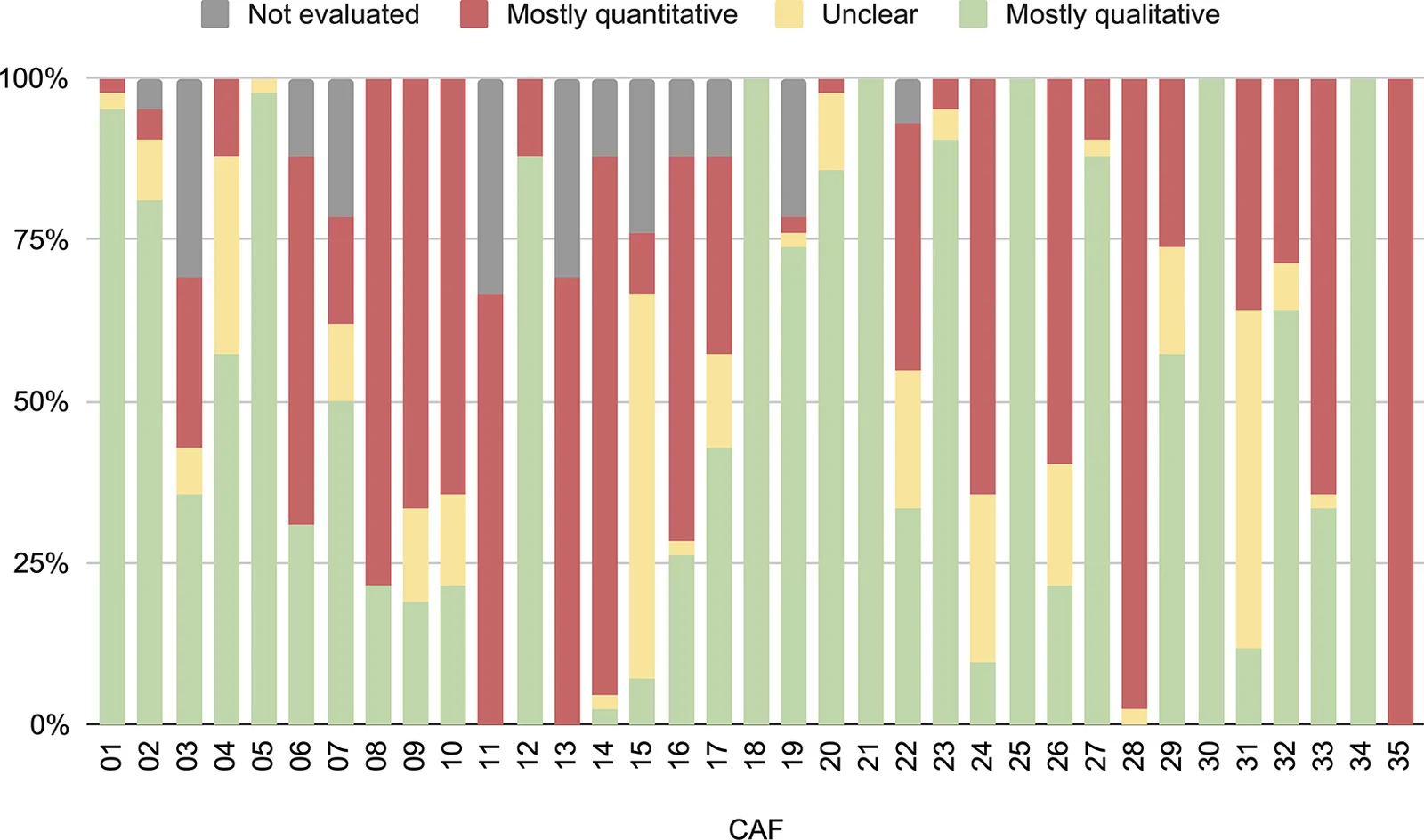
Compliance with Commitment 2 by CAF (Section I) - Senior Researchers.

**Figure 14.**
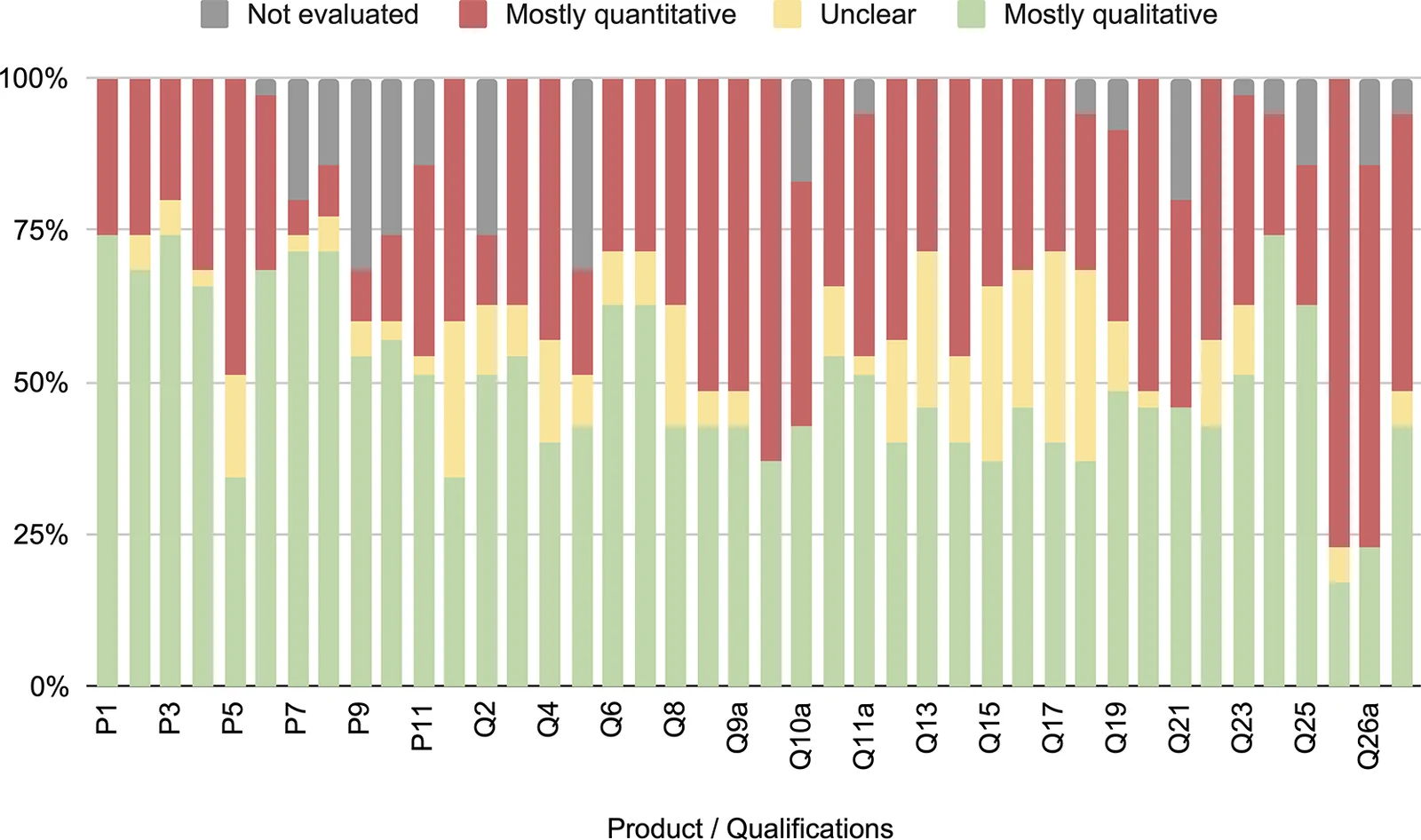
Compliance with Commitment 2 by Product/Qualification (Section I) - Senior Researchers.

Figures 11 to 14 present data on alignment between Commitment 2 and the criteria adopted for Section I (Selected products and qualifications) in the calls for Research Directors and Senior Researchers.

As shown in Table 5 and in Figure 11, just over 57% of CAFs mainly use qualitative criteria, while the remaining 43% are not compliant with Commitment 2.

Among the products, the most quantitatively evaluated are exhibitions, cartographic outputs, concordances, and technical reports. Among the qualifications, coordination of archaeological campaigns, participation in editorial boards, invited lectures, certification activities, organisational positions, scientific and professional qualifications, teaching (including supervision of doctoral students), and evaluation activities stand out.

48.5% of CAFs are compliant with Commitment 2, as more than 50% of the contributions are evaluated using qualitative criteria.

Considering products and qualifications, we observe that 2 product categories (cartographic outputs and concordances) and 2 qualification categories (coordination of, or participation in, archaeological campaigns) receive zero by default in approximately 25% of CAFs, meaning they are neither evaluated nor evaluable.

Moreover, 6 products were consistently evaluated using quantitative criteria in at least 25% of CAFs. These are journal contributions, contributions to volumes, contributions to conference proceedings, databases and software, patents, and technical reports. This applies to all qualifications.

Table 6 presents the analysis related to the calls for technologists (Technologist Directors, Senior Technologists).

**Table 6.** Analysis of evaluation criteria used by the committees against Commitment 2 (Technologist Directors and Senior Technologists).

Call for Technologist Directors	Call for Senior Technologists
** Section I - Analysis by CAF (** Figure 15 **)** 60% of CAFs are fully or mostly compliant with Commitment 2. Conversely, the remaining 40% predominantly rely on quantitative indicators, with over 93% of contributions assessed using such criteria, indicating non-compliance with CoARA Commitment 2. Overall, in 60% of CAFs, strictly quantitative criteria are established in at least one instance. No partially fitting criteria are identified in the calls for Technologist Directors. ** Section I - Analysis by P&Q (** Figure 16 **)** More than 95% of contributions are evaluated primarily using qualitative criteria, and 2.3% are assessed using predominantly quantitative criteria. Except for patents, all research products are evaluated following a mostly qualitative approach in all CAFs. Qualifications are evaluated overall qualitatively. 75.7% of research qualification categories are assessed solely or primarily on qualitative criteria. **Sections II-IV** The criteria established for CV sections II, III, and IV align with Commitment 2.	** Section I - Analysis by CAF (** Figure 17 **)** 80% of CAFs are compliant, as the majority of criteria are based on qualitative judgement. In the other 20%, we note that the criteria follow a mostly qualitative approach for a limited number of contributions. In 40% of cases, partially fitting criteria are identified at least once. ** Section I - Analysis by P&Q (** Figure 18 **)** The criteria established to evaluate research products are overall qualitative in 80% of CAFs. In 40% of CAFs, all research products are assessed using a qualitative approach, and in another 40% patents are the only product to be evaluated based on predominantly quantitative criteria. The trend reverses when we examine the criteria for qualifications. We note a stronger preference for quantitative criteria in all CAFs, except in 20% of them, where nearly 94% of qualifications are assessed using a qualitative approach. Only the following qualifications have partially fitting criteria: Institute Director, Acting Institute Director, Professional Qualifications, and Technical-Administrative Assignments. **Sections II-IV** The criteria established for CV sections II, III, and IV align with Commitment 2.

**Figure 15.**
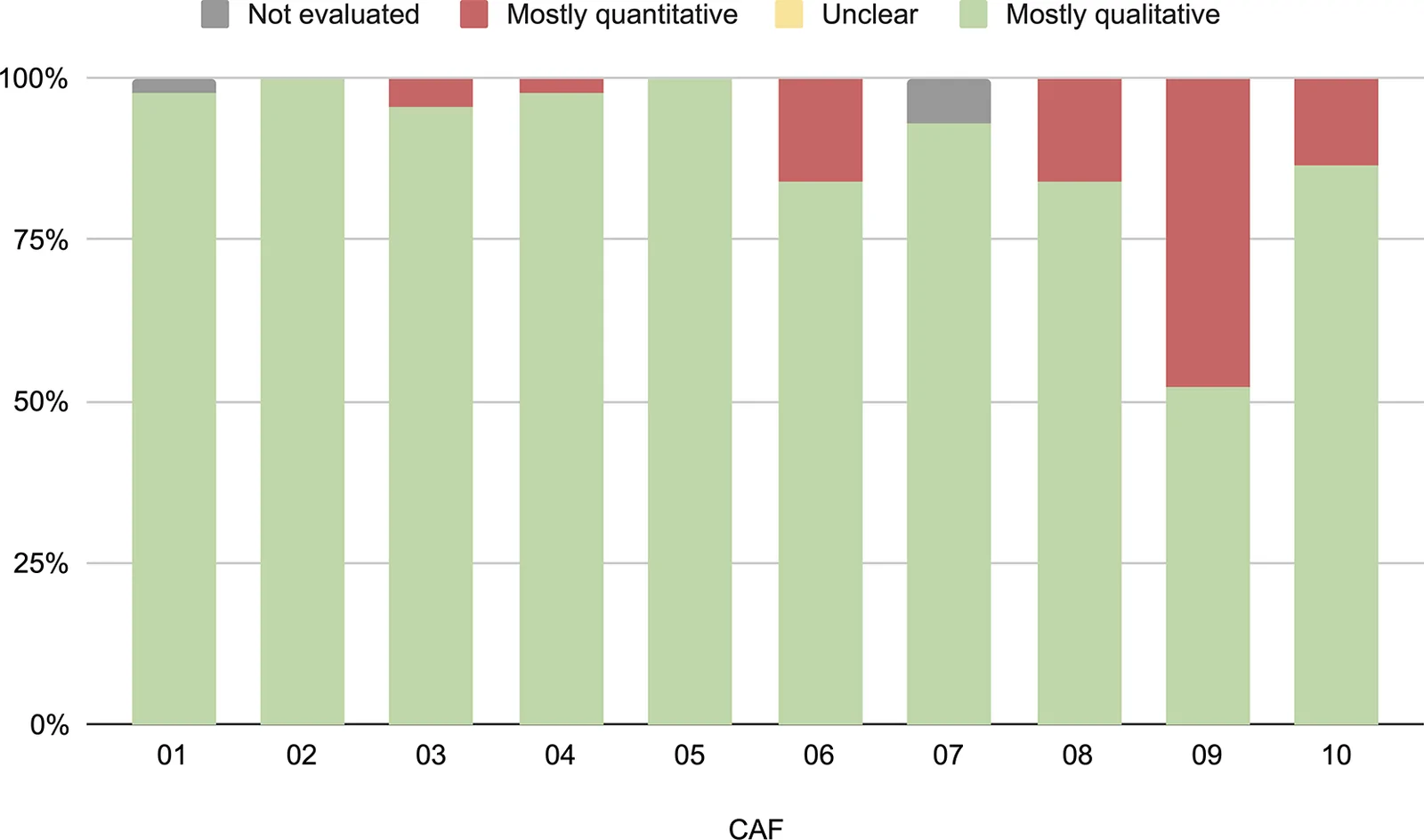
Compliance with Commitment 2 by CAF (Section I) - Technologist Directors.

**Figure 16.**
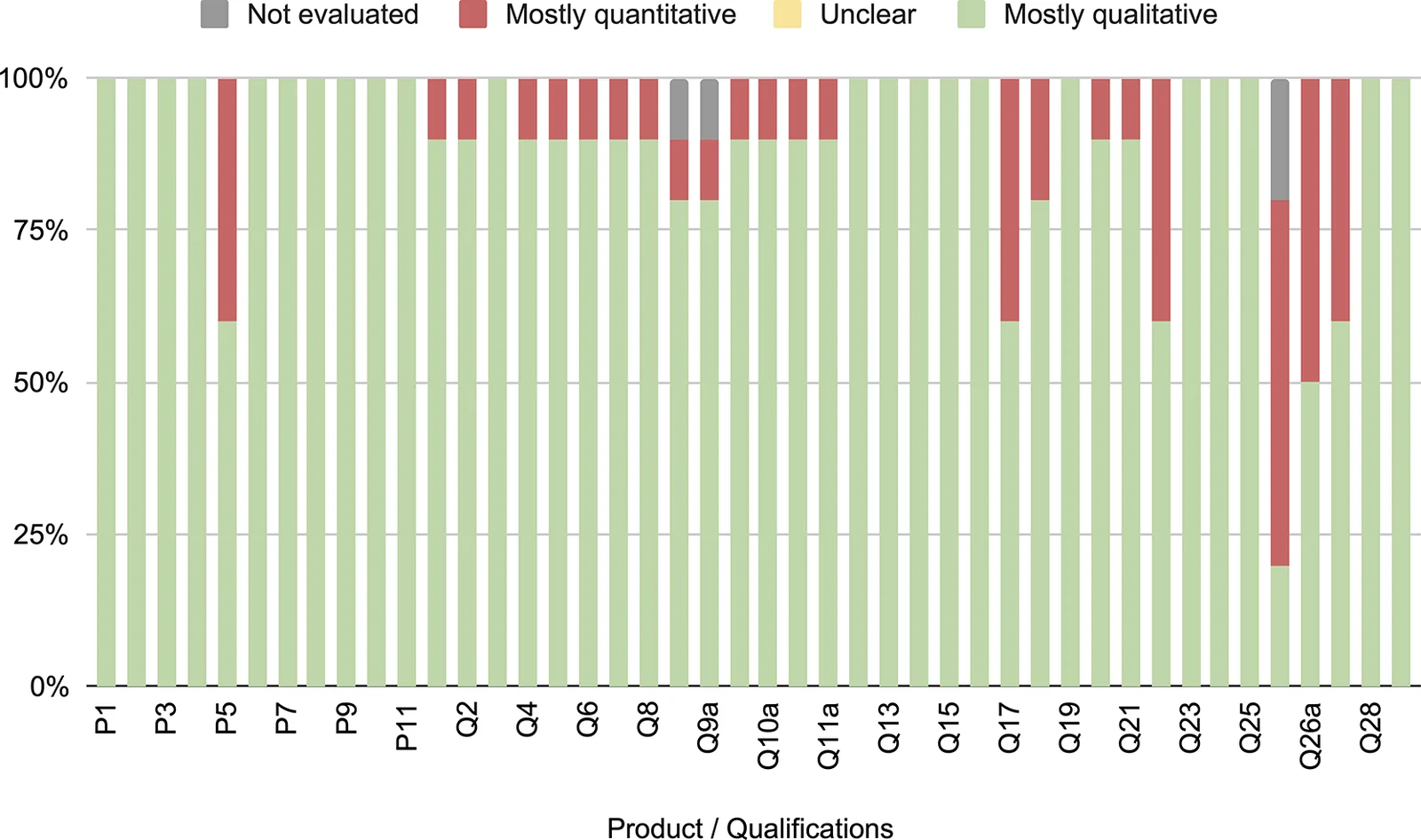
Compliance with Commitment 2 by Product/Qualification (Section I) - Technologist Directors.

**Figure 17.**
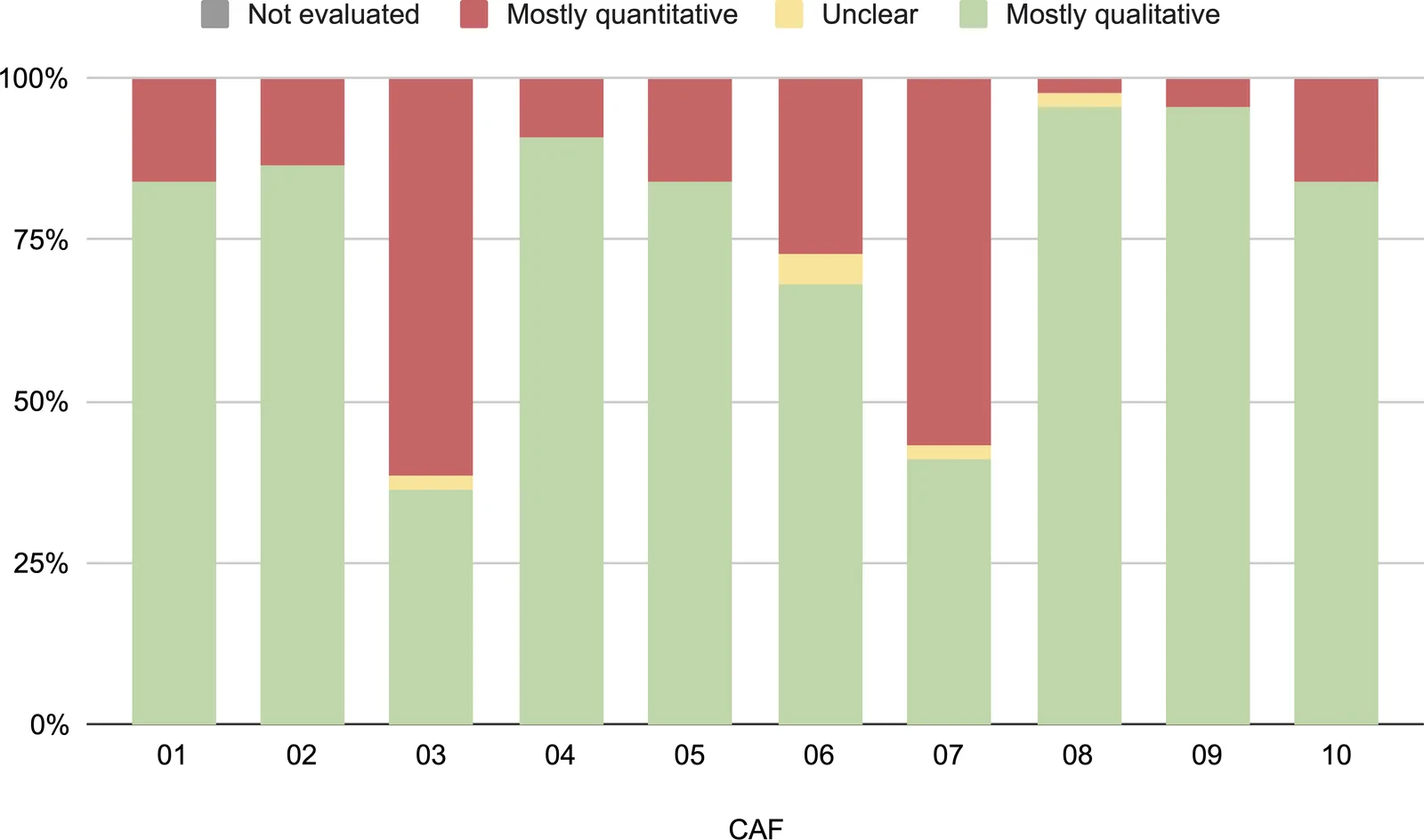
Compliance with Commitment 2 by CAF (Section I) - Senior Technologists.

**Figure 18.**
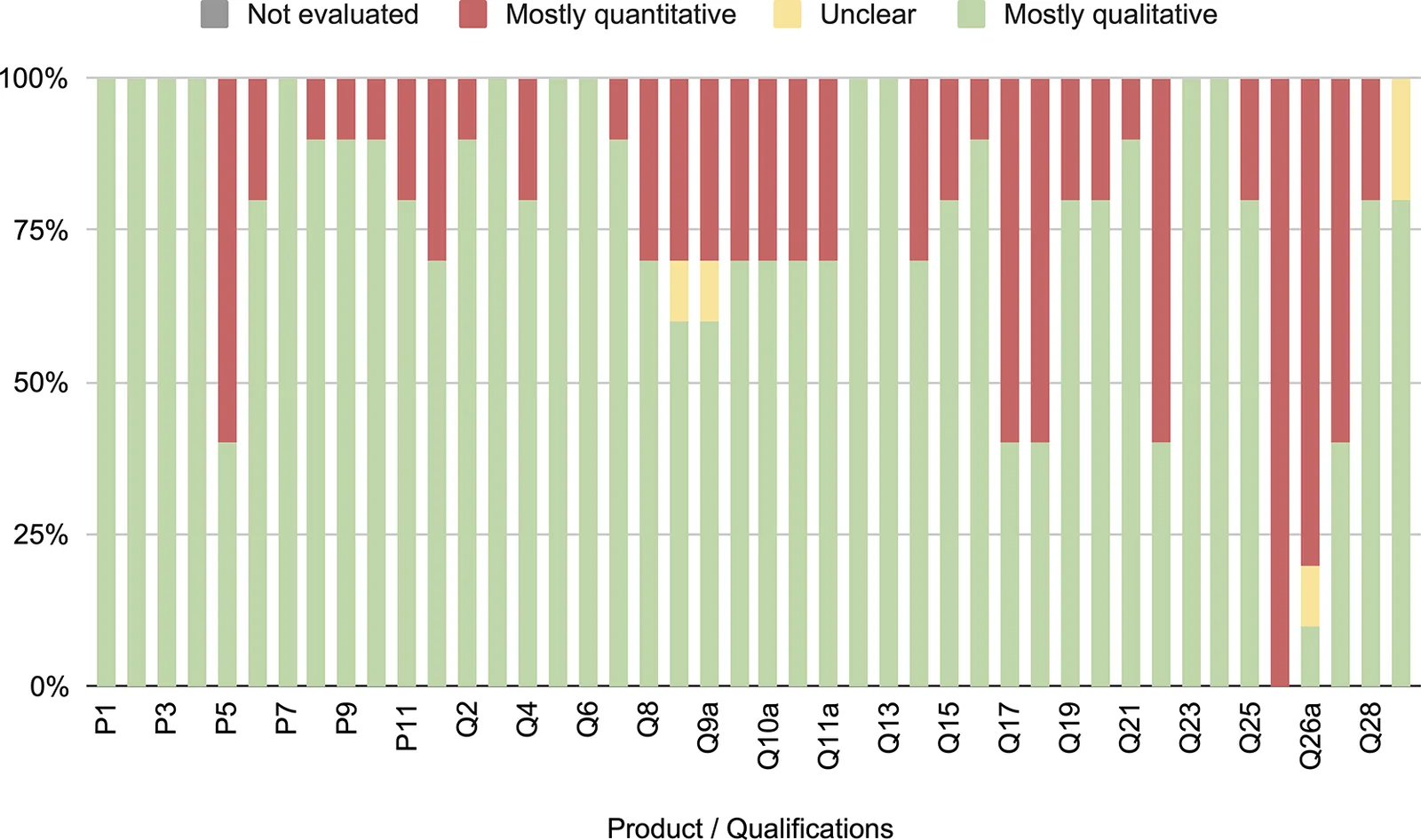
Compliance with Commitment 2 by Product/Qualification (Section I) - Senior Technologists.

Figures 15 to 18 present data on alignment between Commitment 2 and the criteria adopted for Section I (Selected products and qualifications) in the calls for Technologist Directors and Senior Technologists.

As shown in Figure 15, only CAF05 (Management, support and valorisation of research and innovation activities in the biomedical sciences sector) uses solely qualitative criteria, although all CAFs use predominantly qualitative criteria.

Figure 16 shows that patents are assessed using mainly quantitative criteria in over 50% of CAFs. For 6 qualifications, this occurs in over 30% of cases, specifically: awarding of ERC Grants, participation in start-ups, scientific or professional qualifications, and doctoral students supervision. 16 qualifications are assessed using quantitative criteria in over 10% of cases.

While 80% of CAFs are mostly compliant with Commitment 2, we note a stronger preference for quantitative criteria for 1 product category (patent) and for the 6 qualification categories mentioned in the case of Technologist Directors (awarding of ERC Grants, invited lectures, participation in start-ups, scientific or professional qualifications, and doctoral students supervision).

### 5.3 Commitment 3: Abandon inappropriate uses in research assessment of journal- and publication-based metrics, in particular, inappropriate uses of Journal Impact Factor (JIF) and H-index

To analyse compliance with Commitment 3, we focused only on the product criteria, excluding qualifications. Commitment 3 is infringed when research products are evaluated based on the venue, language, or format (i.e., when specific product subcategories are intrinsically valued more than others). Because individual criteria may display multiple forms of infringement, each criterion was counted once in the overall measure of non-compliance, while simultaneously being classified under all applicable categories of infringement. This explains why the percentages reported for specific infringements may exceed the overall proportion of non-compliant criteria.

Table 7 presents the analysis related to the calls for researchers (Research Directors and Senior Researchers).

**Table 7.** Analysis of evaluation criteria used by the committees against Commitment 3 (Research Directors, Senior Researchers).

Call for Research Directors	Call for Senior Researchers
A total of 359 criteria were examined to measure alignment with Commitment 3 ^13^. Of these, 64.3% are compliant, while the remaining 35.7% exhibit one or more misalignment factors. In 15.9% of cases, a different score is assigned to the product based on the **publishing venue**. In disciplines generally evaluated with bibliometric criteria, an explicit mention of Scimago ranking, JIF, quartiles, and indexing in Scopus/WoS is found. In disciplines that are traditionally not evaluated on a bibliometric basis (at least in Italy), we found only one mention of ANVUR classification for A-class venues. In 24% of cases, the maximum possible score varies according to the product’s subcategories (i.e., its format). In 0.6%, scoring differences are noted due to **language**; 40% of the maximum score was deliberately deducted if the product was edited in Italian rather than English. All three factors of misalignment with Commitment 3 are concentrated in the first four product categories (journal contributions, contributions in volumes, books, and conference proceedings), which account for 75.1% of the identified instances of non-compliant criteria. Furthermore, one or more violations of Commitment 3 are present in 82.8% of CAFs.	A total of 347 criteria were examined to measure alignment with Commitment 3 ^14^. Of these, 69.5% are compliant, while the remaining 30.5% exhibit one or more misalignment factors. In 16.7% of cases, products receive different scores depending on their **publication venue**. In bibliometric areas, there are explicit references to the presence of journals in international databases, to Scimago rankings, and to quartiles. In non-bibliometric areas, reference is made to the ANVUR classification (journal indexation and A-class journals). In addition, 19.9% of the criteria are non-compliant because the scores assigned to certain products differ by product subtype, which is the same as relying on publication **format**. A distinction is also noted in the assessment of products in Italian and in English, with a higher score automatically assigned to English- **language** products in 3.2% of criteria. Furthermore, one or more violations of Commitment 3 are present in 82.8% of CAFs.

In over 45% of the criteria in the calls for applications for the two researcher levels, there is a breach of Commitment 3. The format (i.e., the type of product) is the most commonly used criterion, followed by the publication venue and, to a much lesser extent, the language of the contribution. Violations are greater in the first three product categories, namely journal contributions, contributions in volumes, and books, which are generally those that receive the highest potential scores by default. This applies to 82.8% of CAFs.

Table 8 presents the analysis related to the calls for technologists (Technologist Directors and Senior Technologists).

**Table 8.** Analysis of evaluation criteria used by the committees against Commitment 3 (Technologist Directors, Senior Technologists).

Call for Technologist Directors	Call for Senior Technologists
A total of 110 criteria were analysed. Non-compliance with Commitment 3 occurs across 15.5% of criteria, resulting from the use of publication- and format-related metrics to assess certain products. Notably, in 1 CAF, the criteria consistently violate Commitment 3 by assigning decreasing scores according to the type of contribution. Aside from format, we found no discriminatory criteria related to publication venue or language. In general, one or more violations of Commitment 3 are present in 30% of CAFs.	A total of 110 criteria were analysed. Non-compliance with Commitment 3 is observed in 7.3% of criteria. Specifically, in CAF03 and CAF07, inappropriate uses of publication **format** as an evaluation criterion are identified for journal contributions, contributions in volumes, books, and projects. In particular, in both CAFs, while certain subtypes of journal contributions, such as articles, letters, and reviews, can obtain the maximum score of 2 points depending on their scientific quality and the role of the candidate, the remaining subtypes are assigned a fixed score of 0.5 points, regardless of their quality. Similarly, monographs can receive up to 2 points, whereas a fixed score of 0.5 is assigned to other subtypes of contributions, such as edited volumes or translations. No instances are found in this call where a product is evaluated based on its publication venue or language. One or more violations of Commitment 3 are present in 20% of CAFs.

In the calls for proposals for the two technologist levels, there are violations in only 25% of CAFs and 11.3% of criteria, all relating to the publication format, while there are no violations relating to the publication venue or language.

### 5.4 Commitment 4: Avoid the use of rankings of research organisations in research assessment

To assess compliance with Commitment 4, we analysed references to research organisation rankings in the Section I criteria.

Table 9 presents the analysis related to the calls for researchers (Research Directors and Senior Researchers).

**Table 9.** Analysis of evaluation criteria used by the committees against Commitment 4 (Research Directors, Senior Researchers).

Call for Research Directors	Call for Senior Researchers
We identified no explicit usage of rankings of research organisations in the criteria articulated across all CAFs. While the concept of institutional prestige appears in certain CAFs, the criteria do not specify how prestige is defined or applied.	We identified no explicit usage of rankings of research organisations in the criteria articulated across all CAFs. While the concept of institutional prestige appears in certain CAFs, the criteria do not specify how prestige is defined or applied.

Table 10 below presents the analysis related to the calls for technologists (Technologist Directors and Senior Technologists).

**Table 10.** Analysis of evaluation criteria used by the committees against Commitment 4 (Technologist Directors, Senior Technologists).

Call for Technologist Directors	Call for Senior Technologists
We identified no explicit usage of rankings of research organisations in the criteria articulated across all CAFs. While the concept of institutional prestige appears in certain CAFs, the criteria do not specify how prestige is defined or applied.	We identified no explicit usage of rankings of research organisations in the criteria articulated across all CAFs. While the concept of institutional prestige appears in certain CAFs, the criteria do not specify how prestige is defined or applied.

In sum, we did not find any explicit violation of Commitment 4 in any call. However, many refer to the prestige of the institution without defining it.

### 5.5 Open science

In this section, we present the analysis of the compliance between Open Science recognition and the criteria adopted by the evaluation committees, considering a criterion to be compliant if it mentions one or more Open Science practices.

Table 11 presents the analysis related to the calls for researchers (Research Directors and Senior Researchers).

**Table 11.** Analysis of evaluation criteria used by the committees against Open Science recognition (Research Directors, Senior Researchers).

Call for Research Directors	Call for Senior Researchers
** Section I - Selected P&Qs** Open Science practices are mentioned in 9 of the 385 product-related criteria, representing 2.3% of the total. Of these, criteria referring to the public accessibility of software account for 44.4% of the cases. The practices referenced are limited to Open Access (for journal articles, book chapters, projects and prototypes, exhibitions and displays, and technical reports) and Open Source, understood both in its strict sense and as public access to software and databases published in repositories (44.4% of the total cases). Open Science practices are not mentioned in the criteria for qualifications. **Sections II-IV** There is no reference to Open Science practices.	** Section I - Selected P&Qs** Open Science practices are mentioned in 17 of the 385 product-related criteria, representing 4.4%. Of these, 64.7% concern databases and software and address public accessibility of software. The remaining 6 cases concern the following research products: projects and prototypes; exhibitions and displays; cartographic products or maps (1 case); and technical reports (2 cases). The cited practices are limited to Open Access and Open Source, although they always refer only to public access rather than Open Access. Notably, Open Access is never referenced in the criteria for journal contributions, contributions in volumes, books, or conference proceedings. We also note that in many CAFs, penalising scores are assigned to multi-author publications without considering each author’s actual contribution, thereby stifling collaborative research. **Sections II-IV** There is no reference to Open Science practices.

Table 12 presents the analysis related to the calls for technologists (Technologist Directors and Senior Technologists).

**Table 12.** Analysis of evaluation criteria used by the committees against Open Science recognition (Technologist Directors, Senior Technologists).

Call for Technologist Directors	Call for Senior Technologists
** Section I - Selected P&Qs** Open Science practices are mentioned in 5 of 440 product-related criteria, representing 1.1% of the total. Open Science is mentioned in only one CAF, and only in relation to the following research products: databases and software, projects and prototypes, exhibitions, cartographic products, and concordances. It should be noted that public accessibility of the product is stated, rather than using the terms Open Access, Open Data, or Open Source. The concept of Open Science practices is never mentioned in the qualifications section, nor is it referenced in the Narrative CV, sections II-IV. In particular, CAF09 is the only field among the calls for Technologist Directors to exhibit a noticeable, albeit limited, awareness of Open Science practices, a finding consistent with the fact that it is also the sole field explicitly focused on the valorisation and support of research. **Sections II-IV** There is no reference to Open Science practices.	** Section I - Selected P&Qs** Open Science practices are not mentioned in any of the 440 evaluation criteria examined. **Sections II-IV** There is no reference to Open Science practices.

Overall, Open Science practices are mentioned in 0.8% of the criteria for products and qualifications, and in none of the criteria for narrative CVs.

## 6. Discussion and conclusions

Despite the ARRA’s core commitments being explicitly referred to in both the call for applications and the annexes (which have the same legal value), the analysis demonstrates that:

1. The wide variety of research products, processes and activities defining CNR’s mission is largely disregarded. This is demonstrated by breaches of Commitment 1, recorded in 94.4% of cases. In particular, this figure concerns 85% of technologists’ commissions and 98.6% of researchers’ commissions.

For Senior Researchers, none of the committees is fully compliant with Commitment 1, and 34.3% of them explicitly exclude at least one contribution type from the assessment, meaning that a score equal to 0 is assigned by default. For Research Directors, only 5.7% of the committees are compliant, and 48.6% exclude at least one or more products or qualifications. In two cases, up to 31% of the total products and qualifications are considered to have a null value.

In general, infringements of Commitment 1 demonstrate a failure to recognise the elements that define the Institution’s mission, as stated in Article 3 of its Statute, and the self-positioning logic outlined in the CV model. The findings suggest that, although clear efforts to broaden the range of contributions considered in research assessment are observed in a few evaluation committees, inconsistencies remain. Certain recurring contribution types are still undervalued, specifically teaching and tutoring, evaluation activities, as well as science for policy, and public engagement. These activities are all included in Dimension 3, “Technical-scientific support and knowledge dissemination and communication activities”, of the researchers’ self-positioning.

For instance, in the Senior Researchers calls, evaluation activities receive an average of only 1.3 points, which is significantly below the maximum of 3 points. Similarly, participation roles in projects, research infrastructures, and editorial boards are systematically undervalued compared to coordination or responsibility roles. Moreover, evaluation activities are consistently undervalued (having average scores of 1.50 and 1.09 out of 3, with respective standard deviations of 0.542 and 0.535).

Furthermore, significant disparities remain in the recognition of different contributions to and careers in science. Products are, in general, more valued than qualifications, and traditional research products and activities are prioritised over a broader range of contributions. Products that fall outside the “mainstream” types, such as technical reports, exhibitions, cartographic maps, concordances, and conference proceedings, consistently receive lower scores on average, often falling short of half the maximum potential score. This is most evident in the criteria for the two calls addressing researchers, where traditional research products like journal articles are often the only items eligible for the maximum score across nearly all evaluation committees. These patterns indicate that bibliodiversity remains largely absent from the evaluation process.

The situation is less critical in the calls addressing technologists. For example, in the calls for Senior Technologists, 20% of the committees are fully compliant with Commitment 1, and no products or qualifications are entirely excluded from the assessment. Moreover, 77% of research products and 24.1% of qualifications are eligible for the maximum score.

2. **Quantitative (often mechanical) criteria are extensively used at the expense of qualitative ones.** The data show that the second commitment is violated by 47.8% of committees (a figure that is more pronounced in calls for researchers, where it rises to 48.6%, and in calls for Senior Researchers, where it exceeds 54%).

In many of these cases, the adopted criteria are entirely mechanical and are applied regardless of the quality of the product or qualification. For instance, in the Senior Researcher calls, evaluation activities, patents, technical reports, exhibitions, and cartographic maps are frequently scored using rigid numerical indicators, rather than through qualitative assessment. One example is teaching assignments, which are scored on the basis of the number of hours worked (0.1 points per 10 hours of teaching, up to a maximum of 1 point). This approach results in a largely mechanical evaluation, in which the quality or significance of the contribution is not fully considered, reflecting a persistent reliance on quantitative metrics.

In contrast, the evaluation of technologist contributions is better aligned with Commitment 2. In the calls for Technologist Directors, 60% of the committees are mostly compliant with the Commitment. This figure rises to 80% for Senior Technologist calls.

In general, a further qualitative analysis of the criteria adopted for sections II-IV (narrative CV) could be very useful for understanding how committees interpret requests to adopt qualitative judgements and in highlighting the essential features of these interpretations. While these narrative sections were largely compliant with Commitment 2, notable exceptions were found in which committees failed to provide detailed criteria for assigning scores to these sections, leaving the process obscure.

Significantly, a consistent pattern emerges across the first two commitments. Committees tend to apply uneven evaluation criteria across different types of contributions, both in terms of disciplinary preferences and methodological approaches. Although many products and qualifications are not formally excluded from assessment, fixed scoring based on product typology rather than quality weakens both the recognition of diverse career trajectories and the exercise of qualitative judgement. This correlation can be investigated further.

3. **An inappropriate use of metrics based on journals and authors is still present.** The data show that one or more violations of Commitment 3 are present in 28% of the criteria for products, rising to 32.8% in the criteria for Researchers calls. In these cases, the criteria explicitly reference metrics based on journals, format, language, or publication venue.

In the call for Research Directors, where 82.8% of the CAFs adopted at least one criterion non-compliant with Commitment 3, all three factors of misalignment with Commitment 3 are concentrated in the most highly valued product categories (journal contributions, contributions in volumes, books, and conference proceedings) and account for 75.1% of identified instances of non-compliance. In bibliometric-heavy disciplines, committees make explicit references to JIF, Scimago rankings, quartiles, and indexing in Scopus or Web of Science. Even in traditionally non-bibliometric areas, committees reference ANVUR classifications for “A-class” venues. Furthermore, linguistic bias is noticeable: in some cases, a 40% deduction is applied if a product is in Italian rather than English, while in others, a higher score is automatically assigned to English-language products.

In contrast, in the technologist calls, we observe lower levels of non-compliance, equal to 25% of CAFs and the violations relate exclusively to publication format (e.g., assigning fixed lower scores to edited volumes or translations regardless of quality) rather than venue or language.

4. Despite Commitment 4 (avoiding the use of rankings of research organisations) not being directly contradicted in any evaluation committee, some criteria refer to the generic prestige of an organisation. While no formal international university rankings were explicitly used, the concept of institutional prestige appears in certain committees without further clarification on how it is defined or applied. This reliance on vague “prestige” may result in an evaluation process which lacks transparency and consistency, undermining the goal of preventing external ranking methodologies from trickling down into individual researcher assessments.

5. **Open Science practices are generally not recognised or valued**, as they are mentioned in only 1.8% of the criteria relating to products alone, in 0.8% of the criteria relating to products and qualifications, and in 0% of the criteria relating to narrative CVs. This occurs despite the inclusion of metadata related to Open Access for selected products in ‘Attachment C. Selected products and qualifications’ completed by candidates, as well as explicit indications in ‘Attachment B. Professional CV guidelines’ to value collaborations, multilingualism and multidisciplinarity. In addition, in many evaluation committees, penalising scores are assigned to multi-author publications without considering the actual contribution of each author. This approach is detrimental to collaborative research and multidisciplinarity. Finally, the practices that are mentioned remain very limited (primarily Open Access, Open Source, and a generic notion of “public access”) and indicate little or no awareness of the broader scope of Open Science on the part of the committees.

Overall, a significant discrepancy is observed between the criteria set out in the competitive calls and those established by the evaluation committees, particularly with regard to Commitment 1 and the recognition of Open Science. This is a cause for concern, as the persistence of criteria rooted in a pre-reform research culture risks encouraging conservative behaviour among candidates. Evidence suggests that publications, especially those in (top) journals, continue to be perceived as the most reliably rewarded research products, while qualifications remain subject to considerable uncertainty in assessment. In particular, the criteria adopted for Senior Researchers exhibit violations of the first three core commitments more prominently than those applied to Research Directors and Technologists. This contrast is even more striking if contextualised: the calls for Senior Researchers attracted the largest number of participants, accounting for 69% of the total, and 50% of these candidates will participate in the next call.

The data collected here are extensive and rich, allowing the analysis to be expanded in several directions, both to monitor the outcomes of the evaluation process and to better understand the reasons underlying committee decisions.

As mentioned above, a qualitative analysis of the criteria adopted in sections II-IV (relating to the narrative CV) would be useful for understanding how the mandate of Commitment 2 was interpreted in practice in this context.

Furthermore, it would be interesting to analyse the degree of similarity among the criteria adopted by different committees. Our observations suggest the presence of clusters of similar criteria, likely reflecting the gradual and uneven publication of committee criteria over time. This analysis could then be related to the CAF’s disciplinary areas, following a mapping of CAFs onto ERC sectors. In fact, as underlined above, CNR has adopted its own classification system, which does not correspond to either Italian university scientific-disciplinary sectors or ERC ones.

Similarly, another relevant strand of analysis concerns the relationship between breaches of Commitments 1, 2 and 3. Exploring these correlations would help clarify how the different dimensions addressed by the commitments interact in practice.

Finally, it would also be useful to compare the criteria adopted in this career progression process with those used in previous competitions, particularly the 2020 call, in order to assess whether improvements have occurred following the introduction of ARRA commitments. Such an analysis would require mapping the CAFs used in this competition onto those of earlier procedures, which do not always coincide.

In addition, the framework for assessing CoARA compliance devised in this study, although dependent on the context of this assessment, contributes to the discussion about the possible interpretations of the ARRA and its implementation. A discussion to which the CoARA community is called to contribute.

In general, questions remain about whether an assessment reform can be implemented without requiring the committees to comply with the Authority’s high-level criteria. Indeed, there is no clear guidance on which criteria should take precedence in the event of a conflict, as occurred in this instance. An answer may emerge if some candidates decide to appeal the criteria before the courts. In this context, the persistence of misalignments between policy and practice serves as a reminder to mind the gap between formal commitments and actual implementation, as compliance with established policy frameworks alone does not ensure their effective application.

## Ethics and consent

Ethical approval and consent were not required.

## Data Availability

The study reuses publicly available institutional documents issued by the National Research Council of Italy (CNR), including the 2023 competitive calls for career progression (calls 315.62–315.65), regulatory attachments, and evaluation criteria published by disciplinary committees. These documents are available through the CNR Public Relations Office (URP) portal ( https://archivio.urp.cnr.it/page.php?level=3&pg=47&Org=4&db=1). Repository: Zenodo. Underlying data to ‘Mind the Gap: From Policy to Practice at CNR. An Analysis of Career Advancement Criteria in Light of the Agreement on Reforming Research Assessment’ (1.0) [Data set]. Zenodo. https://doi.org/10.5281/zenodo.18657087 ( Di Donato et al., 2026a). The repository contains the following underlying data:
• Data file 1. CNR_Career_Progression_2023_Research_Director_(315.62).xlsx (Structured dataset containing the annotated evaluation criteria for the 2023 CNR call for Research Director positions) • Data file 2. CNR_Career_Progression_2023_Senior_Researcher_(315.63).xlsx (Structured dataset containing the annotated evaluation criteria for the 2023 CNR call for Senior Researcher positions.) • Data file 3. CNR_Career_Progression_2023_Technologist_Director_(315.64).xlsx (Structured dataset containing the annotated evaluation criteria for the 2023 CNR call for Technologist Director positions.) • Data file 4. CNR_Career_Progression_2023_Senior_Technologist_(315.65).xlsx (Structured dataset containing the annotated evaluation criteria for the 2023 CNR call for Senior Technologist positions.) • Data file 5. Readme - Career progression data.rtf (Documentation describing the dataset structure, variables, annotation methodology, and instructions for reuse.) Data are available under the terms of the Creative Commons Attribution 4.0 International (CC-BY 4.0) licence. Repository: Zenodo. Extended data for the article ‘Mind the Gap: From Policy to Practice at CNR. An Analysis of Career Advancement Criteria in Light of the Agreement on Reforming Research Assessment’. https://doi.org/10.5281/zenodo.18923892 ( Di Donato et al., 2026b). This repository contains the following extended data:
• Annex A – Career Advancement Fields for researchers. (Table presenting the Career Advancement Fields used in the CNR evaluation procedures for researchers, including the original Italian terminology and English translations.) • Annex B – Career Advancement Fields for technologists. (Table presenting the Career Advancement Fields used in the CNR evaluation procedures for technologists, including the original Italian terminology and English translations.) •
Annex C – Research products and qualifications for researchers. (Table presenting the research products and qualifications considered in the evaluation procedures for researchers, with Italian terms and English translations.) • Annex D – Research products and qualifications for technologists. (Table presenting the research products and qualifications considered in the evaluation procedures for technologists, with Italian terms and English translations.) • Annex E – Research questions. (Table listing the research questions used in the analysis, organised according to the commitments of the Agreement on Reforming Research Assessment and the recognition of Open Science practices.) • Annex F – List of analysed documents. (Table listing the official CNR calls, attachments, and evaluation criteria analysed in this study.) • Annex G - Glossary of key terms used in the study (Glossary of key Italian terms used in the CNR career advancement procedures.) Extended data are available under the terms of the Creative Commons Attribution 4.0 International (CC-BY 4.0) licence.

## Footnotes

^1^
As of 31/12/2024. Of these, around 58% are researchers, 12% are research technologists, 21% are technicians, and 9% are administrative staff.

^2^

CNR Statute, art. 3 (objectives) defines all elements of the CNR mission, 
https://www.cnr.it/sites/default/files/public/media/STATUTES%20OF%20THE%20CONSIGLIO%20NAZIONALE%20DELLE%20RICERCHE.pdf.

^3^
The professional profiles are defined by the national guidelines ( Gazzetta Ufficiale, 1991).

^4^
As of 21 January 2026, https://coara.eu/agreement/signatories/.

^5^
GraspOS project, https://graspos.eu (Grant Agreement 101095129).

^6^
Legislative Decree 218/2016. In direct appointment procedures, it is mandatory to obtain the opinion of the Scientific Council and the approval of the Board of Directors.

^7^
Guidelines for researchers (Senior and Directors) and for technologists (Senior and Directors) are slightly different.

^8^

https://selezionionline.cnr.it

^9^

https://github.com/andremann/graspos-pilot-cnr

^10^
42 criteria * 35 CAFs * 2 researcher selections = 2,940 criteria plus 44 criteria * 10 CAFs * 2 technologist selection = 880 criteria, plus criteria for valorisation sections II-IV*90 CAFs.

^11^
CNR profiles explicitly mention international recognition as a key aspect in the profiles of Research and Technologist Directors, while this is not reported for Senior Researchers and Technologists.

^12^
For each call, a dedicated spreadsheet is provided. Each spreadsheet reflects our annotations regarding the compliance with the four ARRA commitments and the presence of Open Science-related criteria for each research product and qualification (and, where relevant, for Sections II-IV criteria) and CAF. Further details, such as the colour coding adopted in the annotation phase, are reported in the README file of the dataset.

^13^
From the total of 385 criteria, we subtracted the 26 which receive 0 points by default. These cases are not included in our analysis of compliance with CoARA Commitment 3, leaving a total of 359 criteria to analyse.

^14^
From the total of 385 criteria, we subtracted the 38 which receive 0 points by default. These cases are not included in our analysis of compliance with CoARA Commitment 3, leaving a total of 347 criteria to analyse.

## Software availability

The code is licensed under the MIT License (OSI-approved).

Repository: Zenodo. Raw data for the implementation of the GraspOS CNR Pilot. The repository contains the Python script used to collect the source PDF documents from the CNR portal. https://doi.org/10.5281/zenodo.18649943 ( Mannocci, 2026).

The code is also available for version control and reuse on GitHub: https://github.com/andremann/graspos-pilot-cnr
