# Supplementary material for: Mind the Gap: From Policy to Practice at CNR An Analysis of Career Advancement Criteria in Light of the Agreement on Reforming Research Assessment
Source: Open Res Eur. 2026 Jul 23;6:127. Originally published 2026 Apr 19. [Version 2] doi: 10.12688/openreseurope.23373.2 (PMC13554998; doi:10.12688/openreseurope.23373.2)
Supplement: Peer review history

# Reviewer report: Reviewer response for version 2

**DOI:** 10.21956/openreseurope.26530.r77912

**Published:** 2026-08-05

**Authors:** Isabella Peters

**Affiliations:** 1 ZBW Leibniz-Informationszentrum Wirtschaft & Universität Kiel, Kiel, Germany

Dear authors, thank you for revising the text and adressing my comments in the article as well as in the letter of response. You sufficiently clarified what I have found a bit difficult to understand.

If applicable, is the statistical analysis and its interpretation appropriate?

I cannot comment. A qualified statistician is required.

Is the study design appropriate and is the work technically sound?

Yes

Is the work clearly and accurately presented and does it engage with the current literature?

Yes

Are the conclusions drawn adequately supported by the results?

Yes

Are sufficient details of methods and analysis provided to allow replication by others?

Partly

Are all the source data and materials underlying the results available?

Yes

Reviewer Expertise:

I have more than 15 years of expertise in research evaluation, scholarly metrics, and the intersection of scholarly communication and open science—all of which are central to the themes of this paper.

I confirm that I have read this submission and believe that I have an appropriate level of expertise to confirm that it is of an acceptable scientific standard.

# Reviewer report: Reviewer response for version 2

**DOI:** 10.21956/openreseurope.26530.r77913

**Published:** 2026-07-25

**Authors:** Lex Bouter

**Affiliations:** 1 Amsterdam Universities Medical Center, Amsterdam, North Holland, The Netherlands; 2 Philosophy, Vrije Universiteit Amsterdam (Ringgold ID: 1190), Amsterdam, North Holland, The Netherlands

The authors made a number of adequate improvements in response to my suggestions, adding a section on study limitations being the most important of them. Unfortunately they decided not to shorten the manuscript substantially, which in my view means that most readers won't bother to struggle through the over-detailed narrative. That will decrease the impact of the study substantially I fear. I was also a bit disappointed in the tone of the rebuttal ('we already explained that in the initial manuscript, but let us stipulate the details once more') and the lack of clear information about what was changed where in the revised manuscript.

 But - everything taken together - for me the revised manuscript is good enough to be approved for indexing.

If applicable, is the statistical analysis and its interpretation appropriate?

Not applicable

Is the study design appropriate and is the work technically sound?

Partly

Is the work clearly and accurately presented and does it engage with the current literature?

Partly

Are the conclusions drawn adequately supported by the results?

Partly

Are sufficient details of methods and analysis provided to allow replication by others?

No

Are all the source data and materials underlying the results available?

Yes

Reviewer Expertise:

methodology, research integrity, open science, meta-research, research ethics

I confirm that I have read this submission and believe that I have an appropriate level of expertise to confirm that it is of an acceptable scientific standard.

# Response

**Published:** 2026-07-30

**Authors:** Lottie Provost

**Affiliations:** Istituto di Linguistica Computazionale “A. Zampolli”, Consiglio Nazionale delle Ricerche, Italy

We thank Lex Bouter for his careful engagement with the manuscript across both rounds of review, and for his approval of the revised version for indexing. On the point about version tracking, we would like to note that a summary of changes between V1 and V2 was included in the "Amendments from Version 1" section accompanying the revised manuscript. Thank you again for the constructive feedback which helped us improve this work.

# Reviewer report: Reviewer response for version 1

**DOI:** 10.21956/openreseurope.25286.r74927

**Published:** 2026-07-09

**Authors:** Isabella Peters

**Affiliations:** 1 ZBW Leibniz-Informationszentrum Wirtschaft & Universität Kiel, Kiel, Germany

**1. Summary of article**

 In 2023, the National Research Council of Italy (CNR) launched four competitive calls aimed at the career advancement of researchers and technologists, operationalizing the principles and commitments of the Agreement on Reforming Research Assessment (ARRA) as a novel evaluation framework. This study examines the alignment between the criteria established by the evaluation committees and the ARRA’s core commitments. This is the first detailed analysis of the actual implementation of ARRA commitments in career advancement calls at the institutional level of a national research-performing organization, assessed in relation to its strategic orientations and formal policies. The key finding of the study includes: The implementation of ARRA commitments requires improvement, as all four commitments are only partially addressed. A significant discrepancy emerges between the criteria outlined in the competitive calls and those applied by the evaluation committees, with Commitment 1 being particularly underrepresented.

**2. Presentation of work and engagement with literature**

 The article presents its work clearly and engages thoroughly with the relevant literature. A minor point of critique refers to the presentation of results in the figures: frequently, the authors merely state that a result is illustrated in a figure without specifying the figure number, which would facilitate reader navigation. Additionally, while the figures often display only the numerical references to CAFs (as far as I understand), the discussion refers to categories such as “Historical sciences for the study of cultures and civilizations in a global comparative perspective” and “Philosophical, linguistic, philological-literary sciences and their digital applications” without providing the corresponding numerical information. This omission complicates the task of locating and verifying the specific results under discussion.

**3. Study design and technical soundness**

 The study design is technically robust, and the methodology is well- documented, rendering it a valuable reference for future research and projects with analogous objectives. The approach appears particularly well-suited for recurring assessments of the effectiveness and comprehensiveness of policy guideline implementation. However, one may question the extent to which meaningful results can already be expected in the case of CNR, given the brief period elapsed since the policy’s adoption and the subsequent need for its practical integration. It is possible that the new guidelines have not yet been fully internalized by the evaluation panel. Furthermore, the degree of awareness among panel members regarding the new policies, as well as the extent of training they have received to ensure their proper application, remains unclear. I hope the authors can shed some more light on this aspect or address it in their “discussion section”.

**4. Details of methods and analysis**

 Overall, the paper provides sufficient detail regarding the methods employed. Nevertheless, it would greatly enhance the reader’s understanding—and serve as a valuable contribution—if the authors would illustrate their assessment process with a concrete example. Without such an example, the methodology can be challenging to follow, particularly when cross-referencing the supplementary material, which is essential for transparency and comprehension. Additionally, it remains unclear whether the authors’ analysis was limited to the evaluation criteria and scores provided in the official documents, or if they also had access to the actual applications (though I assume the latter was not the case).

 I have also two further questions regarding the evaluation process at CNR, as illustrated in Fig. 2:

1. Is it possible for the applicants to change the P&Qs, which were submitted in the first step, after they get informed about the evaluation criteria?
2. Does the evaluation panel screen the submitted P&Qs before they come up with the evaluation criteria or do panel members independently come up with the evaluation criteria?

**5. Availability of source data and materials**

 Source data and material is available on Zenodo and also on GitHub. The documents on Zenodo also include a link to a website ( https://archivio.urp.cnr.it/ ) which contains all PDFs of calls and evaluation criteria. Since websites and URLs may vanish, I recommend sharing the full material (all PDFs) on Zenodo. (I only discovered later that the PDFs are accessible via GitHub – but this was not clear from the text and could be revised since now there are several instances where data, documents and code can be found, and which might be confusing.

**6. Conclusions supported by results**

 The authors introduce a significant methodology for monitoring and benchmarking institutional progress in policy implementation. To enhance the transparency of the evaluation mechanics at CNR, it would be valuable if the authors could clarify which authority determines the admissibility of scores for evaluation criteria—and, by extension, the rationale behind the unequal weighting of these criteria.

 Regarding the observation that “the evaluation of technologist contributions is better aligned with Commitment 2,” the study results indicate that calls for technologists consistently demonstrate stronger alignment with the commitments. Why do you think this is the case? I would appreciate reading the authors’ perspective on the underlying reasons for this finding.

 Given the assumption that the authors lacked access to both the applications and the final scoring decisions of the evaluation committee, I recommend emphasizing in the conclusions that the research evaluation exercise is inherently influenced by the interpretative frameworks of the committee panels. This raises the question: Is there any oversight mechanism at CNR to ensure adherence to ARRA principles?

 Finally, I would like to address a point of curiosity unrelated to the study’s core findings. The authors highlight that “contributions in conference proceedings receive 0.5 points in the Humanities but 2 points in ICT,” and suggest that this divergence contradicts Attachment B of the call, which stipulates that CVs should be assessed independently of the disciplinary area to foster multidisciplinary.” However, it is unclear to me why the authors view this divergence as problematic. Does this not align with ARRA Commitment 1, which explicitly acknowledges that the spectrum of valued research outputs may vary across disciplines? Should not each discipline retain the autonomy to determine the importance of its outputs?

If applicable, is the statistical analysis and its interpretation appropriate?

I cannot comment. A qualified statistician is required.

Is the study design appropriate and is the work technically sound?

Yes

Is the work clearly and accurately presented and does it engage with the current literature?

Yes

Are the conclusions drawn adequately supported by the results?

Yes

Are sufficient details of methods and analysis provided to allow replication by others?

Partly

Are all the source data and materials underlying the results available?

Yes

Reviewer Expertise:

I have more than 15 years of expertise in research evaluation, scholarly metrics, and the intersection of scholarly communication and open science—all of which are central to the themes of this paper.

I confirm that I have read this submission and believe that I have an appropriate level of expertise to confirm that it is of an acceptable scientific standard, however I have significant reservations, as outlined above.

# Response

**Published:** 2026-07-30

**Authors:** Lottie Provost

**Affiliations:** Istituto di Linguistica Computazionale “A. Zampolli”, Consiglio Nazionale delle Ricerche, Italy

We thank Isabella Peters for her careful reading. As V2 has already completed peer review, we respond to her comments directly here. 

1. Figures and CAF references.

The numerical CAF identifiers for the two SSH fields mentioned by name in the manuscript are CAF33 “Historical sciences for the study of cultures and civilizations in a global comparative perspective” and CAF35 “Philosophical, linguistic, philological-literary sciences and their digital applications”.  

1. Timing and committee training

We thank the reviewer for this observation, which we have addressed in the Limitations section added to the revised manuscript (V2). Specifically, we note that the 2023 calls analysed here are the first career advancement calls issued by CNR following its signature of the ARRA in November 2022, and that future calls may reflect greater alignment with ARRA commitments as the culture of research assessment reform continues to evolve. A comparison with future procedures would undoubtedly be a valuable next step. We also clarify in the revised manuscript that panel members' internal preparation, training, and awareness of the new policies fall within the internal deliberation procedures of evaluation committees, which are not documented in the publicly available materials analysed in this study and which we could not access.

1. Concrete example

To illustrate how our coding logic was applied in practice for both Commitment 1 and Commitment 2, we hereby provide a practical application of our methodology focusing on the criterion for university teaching assignments (Q10a) in the Senior Researchers call for CAF35 (Philosophical, linguistic, philological-literary sciences and their digital applications).  This criterion awarded 0.1 points for every 10 hours of teaching, while limiting the maximum score obtainable to 1 point. This cap was considered non-compliant with ARRA Commitment 1, which aims to broaden the recognition of the diverse practices, activities and careers in research. Full compliance would require that all products and qualifications remain eligible for the maximum score of 3. This assessment is documented in cell AL24 of the "Section I - Commitment #1" sheet within our publicly available Data file 2 on Zenodo.  The same criterion also triggered a non-compliance flag for ARRA Commitment 2, but for a different reason: the score was determined through a purely quantitative measure based on the number of teaching hours, rather than through a qualitative assessment of the content, relevance, or significance of the teaching contribution. By answering "Yes" to the methodological guiding question of whether the qualification could be assessed without examining its content, the criterion was classified as predominantly quantitative. These findings are recorded in cell AL24 of the "Section I - Commitment #2" sheet of the same dataset. 

1. Scope of analysis

We confirm the reviewer's assumption: The calls and the attachments have been collected by accessing the official documents published on the CNR’s public relations office portal. The analysis was based exclusively on these published calls, attachments, and committee-defined criteria, as we did not have access to individual candidate applications and committees' final scores or ranking decisions. 

1. Process questions (Figure 2)

Can applicants revise P&Qs after criteria are published?

 During the application phase, candidates are required to select a preliminary set of products and qualifications for CV Section I. Once the evaluation committees publish their criteria for each CAF, candidates are given the opportunity to select a short number of products and qualifications and finalise their CV Section I before the committees proceed to evaluate the applications.  Do committees see submitted P&Qs before setting criteria?

 Although candidates' products and qualifications are submitted before the evaluation committees are formally nominated, committees do not have access to individual applications when formulating their evaluation criteria. Criteria are established solely with reference to the call for applications, its regulatory attachments, and the list of eligible product and qualification types, independently of the content of any specific candidate's submission.  

1. Source data preservation

Indeed, the archived source PDFs and collection code are on Zenodo via Mannocci (2026), https://doi.org/10.5281/zenodo.18649943 (mirrored on GitHub). 

 For clarity: source PDFs and code are to be found in Mannocci (2026); annotated criteria dataset in Di Donato et al. (2026a); and extended data and annexes in Di Donato et al. (2026b).

1. Score admissibility and weighting authority

Per CNR's regulatory framework, committees are independent in composition and in setting criteria. Once constituted, no central authority approves or reviews their specific scoring weights before publication. The call fixes only the overall point totals per section (Table 2); everything below that is committee discretion. 

1. Why technologists show stronger Commitment 2 alignment

The stronger Commitment 2 alignment in technologist calls appears closely linked to the nature of technologist CVs. In researcher calls, non-compliant quantitative scoring is concentrated on outputs most easily assessed through existing bibliometric proxies. Technologist CVs, by contrast, focus more on patents, technical reports, databases/software, and infrastructure or project coordination, for which no equivalent standardised metrics exist. Except for patents, committees therefore had little choice but to engage qualitatively with the substance of the contributions.

1. Interpretative frameworks and oversight

Indeed, because committees are independent once constituted and criteria-setting is left to their discretion, our results necessarily reflect each committee's own interpretation of the ARRA commitments rather than a centrally-verified application of them. There is no dedicated mechanism at CNR to review committee criteria against ARRA principles before publication.

1. Differences in scoring the same contribution across disciplines

We agree Commitment 1 explicitly allows and encourages disciplines to value different types of output differently, and we are not suggesting that disciplinary variation in scoring conference proceedings is itself a violation of Commitment 1. Our concern is narrower: Attachment B specifically instructs committees to assess certain CV elements independently of the CAF's own disciplinary area, in order to recognise and reward multidisciplinary engagement. The divergence we highlight is noteworthy because it sits in tension with that specific procedural instruction, not because disciplinary autonomy over valuing outputs is inherently problematic.

# Reviewer report: Reviewer response for version 1

**DOI:** 10.21956/openreseurope.25286.r74923

**Published:** 2026-06-24

**Authors:** Lex Bouter

**Affiliations:** 1 Amsterdam Universities Medical Center, Amsterdam, North Holland, The Netherlands; 2 Philosophy, Vrije Universiteit Amsterdam (Ringgold ID: 1190), Amsterdam, North Holland, The Netherlands

The manuscript reports on an evaluation of 4090 criteria formulated by 90 committees installed by the National Research Council of Italy (CNR - 88 institutes – 9707 employees) for handling the 2023 competitive calls for career progression of employees in CNR’s disciplinary clusters. The evaluation concerns desk research, in which the compliance is assessed of these career progression criteria with 4 commitments of the Coalition for advancing Career Assessment (CoARA) and Agreement on Reforming Research Assessment (ARRA) guidance, to which the CNR has committed herself. The conclusion is quite simple: despite all good intentions, the CoARA and ARRA guidance trickles down in the criteria only to a quite limited extent. More specifically: bibliometric indicators based on publication and citation counts still dominate, and open science practices are only rarely recognized by the formulated criteria.

 The manuscript is unusually long (29 pages, 18 figures, 12 tables) and is rather difficult to follow for readers who are not familiar with the CNR policies and procedures. It took me a while to grasp the main points of what was investigated and I’m far from sure whether I succeeded. To make the manuscript easier to follow for uninitiated readers, I believe two things need to happen:

 1. Production of a much shortened revised manuscript of let’s say maximally 10 pages, 2-3 tables and 2-3 figures, and

 2. Reformulation of the narrative in a much less detailed way and with a stronger focus on its main points, while taking into account the major issues listed below.

 I would like readers to get a clear view on the ‘big picture’, without being distracted by so many details. These details of course do matter and need to be documented, but should be transferred to digital supplements in other not to confuse.

- Despite (or maybe because of) all the details provided, I still found it difficult to understand how decisions on career progression are made within CNR. There seem to be regular calls for employees (or their superiors?) to submit an application and then these applications are judged by a dedicated committee in the light of a set of criteria they formulated for the function type and disciplinary cluster they are responsible for. What I found unclear:
  1. Do these criteria have different weights and how were these weights established?
  2. On what is the scoring based: e.g. Curriculum Vitea and publication list, company records, judgement of superiors?
  3. Who is doing the scoring: e.g. generative AI, HRM staff or committee members?
  4. What do we know about the inter- and intra-rater agreement of the scoring? Are there procedures for reaching consensus if raters disagree? Are scores of raters normalized (to remove systematic differences between the raters)?
  5. Assuming that the scoring leads to a ranking of candidates, what does that lead to: e.g. invitation for an interview of the X highest scoring candidates, all candidates above a cut-off score, etc.?
  6. Assuming that interviews are held, are the scores or rank order integrated in the final decision (career progression [promotion] or not)? And if so, how are score and interview impression combined?
- Please explain better why readers should be interested in this Italian case history. My impression is that in international perspective such an amazingly granulated policy and procedure for career progression is quite rare. What can others learn from your in-depth exploration of this single case?
- Please openly recognize that you’re studying an intermediate outcome (how good do the assessment criteria reflect the policy commitments of CNR?). You are not studying the real endpoint: do the career progression decisions reflect the CNR adoption of the CoARA and ARRA commitments? In my experience with academic assessment committees the formal criteria play only a limited role in their decisions. So I’m not convinced that your intermediate outcome is a good predictor of the final endpoint. Please explain why you didn’t study that final endpoint explicitly.
- The criteria the CNR career progression committees used in 2023 were evaluated in this project. Please reflect on what has changed since. This might be important, because the culture change in researcher assessment seems to have accelerated substantially over the last few years.
- In the light of the last three issues listed above, I find it unacceptable that a study limitation section is missing from the manuscript.

If applicable, is the statistical analysis and its interpretation appropriate?

Not applicable

Is the study design appropriate and is the work technically sound?

Partly

Is the work clearly and accurately presented and does it engage with the current literature?

Partly

Are the conclusions drawn adequately supported by the results?

Partly

Are sufficient details of methods and analysis provided to allow replication by others?

No

Are all the source data and materials underlying the results available?

Yes

Reviewer Expertise:

methodology, research integrity, open science, meta-research, research ethics

I confirm that I have read this submission and believe that I have an appropriate level of expertise to state that I do not consider it to be of an acceptable scientific standard, for reasons outlined above.

# Response

**Published:** 2026-07-03

**Authors:** Lottie Provost

**Affiliations:** Istituto di Linguistica Computazionale “A. Zampolli”, Consiglio Nazionale delle Ricerche, Italy

We thank the Reviewer for their time and detailed feedback. We believe that several of the points raised are already addressed in the current version of the manuscript. We nevertheless welcome the opportunity to clarify them and hope that the responses below help move the revision process forward. Reviewer comment (Q1)

*The manuscript is unusually long (29 pages, 18 figures, 12 tables) and is rather difficult to follow for readers who are not familiar with the CNR policies and procedures. It took me a while to grasp the main points of what was investigated and I’m far from sure whether I succeeded.* *To make the manuscript easier to follow for uninitiated readers, I believe two things need to happen:* *1. Production of a much shortened revised manuscript of let’s say maximally 10 pages, 2-3 tables and 2-3 figures, and* *2. Reformulation of the narrative in a much less detailed way and with a stronger focus on its main points, while taking into account the major issues listed below.* *I would like readers to get a clear view on the ‘big picture’, without being distracted by so many details. These details of course do matter and need to be documented, but should be transferred to digital supplements in other not to confuse.*

 Response (R1)

 We thank the reviewer for raising this point and agree that readability for an audience unfamiliar with CNR's internal policies and procedures is an important concern. Regarding the request to reduce the manuscript to a maximum of 10 pages, 2–3 tables, and 2–3 figures, we respectfully do not believe that this is the appropriate solution, while still taking concrete steps to improve readability. The manuscript documents a single, multifactorial analysis of CNR's career-evaluation process. The figures and tables are not illustrative add-ons but the evidentiary backbone of the analysis, documenting the compliance of all 90 evaluation committees across ARRA commitments and calls. Reducing them would require omitting key dimensions of the analysis or replacing them with prose summaries, which would increase rather than reduce complexity and would reduce transparency and replicability. We also considered splitting the study into multiple shorter articles but concluded that this would fragment an interdependent analysis. The four ARRA commitments are evaluated against the same dataset and committees, and the cross-cutting patterns reported in the Discussion depend on maintaining the full structure. That said, we recognise that the reviewer’s core concern is accessibility, and we address this further in the responses below.

 Reviewer comment (Q2)

*Despite (or maybe because of) all the details provided, I still found it difficult to understand how decisions on career progression are made within CNR. There seem to be regular calls for employees (or their superiors?) to submit an application and then these applications are judged by a dedicated committee in the light of a set of criteria they formulated for the function type and disciplinary cluster they are responsible for. What I found unclear: do these criteria have different weights and how were these weights established?*

 Response (R2)

 As indicated in Figure 2, which reads "Candidates apply using the CV structure", candidates autonomously submit their own applications. There is no mention of superiors submitting on their behalf. The weighting of criteria operates at two levels. At the institutional level, CNR centrally established the total points available for each CV section through the calls and Attachment A (summarised in Table 2 of the manuscript). Importantly, the calls explicitly mention the ARRA core commitments, and Attachment A directly references the CoARA agreement signed by CNR in 2022, highlighting the main changes compared to previous selection processes. The ARRA commitments were therefore incorporated into the regulatory documents governing the calls themselves. Within that framework, each of the 90 committees had considerable autonomy to assign maximum scores to individual products and qualifications, effectively determining their relative weight in the assessment. A product assigned a maximum of 0.2 points by a given committee is therefore valued far less than one assigned a maximum of 3 points. These weights were not centrally prescribed by CNR beyond the section-level totals; each committee independently defined them for its CAF. We would like to note that this two-level structure is described in Section 3 of the manuscript, and that the variation in how committees weighted different contributions across CAFs is a central finding of the study, documented extensively in Figures 3–10 and Tables 3–6. The fact that committees set weights that often contradict the ARRA commitments explicitly referenced in the calls makes the gap between policy and practice all the more striking and is central to the paper's argument.  We believed that the submitted manuscript adequately addressed these details, but we edited further the section to make this two-level weighting structure more explicit.

 Reviewer comment (Q3)

*On what is the scoring based: e.g. Curriculum Vitae and publication list, company records, judgement of superiors?*

 Response (R3)

 Scoring is based entirely on the candidate’s submitted application, which consists of four CV sections: selected products and qualifications (Section I), narrative CV (Section II), perspectives and potential (Section III), and professional trajectory (Section IV). In some profiles, seniority and an oral examination also contribute, as indicated in Table 2.  There is no input from company records, endorsement or judgment of superiors; the process is a public competition based solely on the candidate's self-submitted materials, as required by Italian constitutional law governing public-sector recruitment.

 Reviewer comment (Q4)

 Who is doing the scoring: e.g. generative AI, HRM staff or committee members?

 Response (R4)

 The scoring is performed by dedicated evaluation committees nominated by CNR, as described in Section 3 of the manuscript. These committees are composed of members subject to a legal requirement for the prevalence of external (independent) members, as established by Decree-Law DL 127/2003. No AI tools, HRM staff, or superiors are involved in the scoring process. We made this clearer in the revised manuscript.

 Reviewer comment (Q5)

 What do we know about the inter- and intra-rater agreement of the scoring? Are there procedures for reaching consensus if raters disagree? Are scores of raters normalised (to remove systematic differences between the raters)?

 Response (R5)

 We thank the reviewer for raising these important methodological questions. However, they fall outside the scope of our study, which focused exclusively on the publicly available evaluation criteria rather than the committees' internal working procedures. The internal deliberation procedures of evaluation committees, including how the workload is shared, how individual scores are aggregated, how disagreements are resolved, and whether scores are normalised across committee members (i.e., raters), are not documented nor released in the publicly available materials we analysed. Investigating the actual scoring process would require access to internal committee documentation, which is strictly confidential. We explicitly acknowledged this as a limitation in the dedicated limitations section that we added to the revised manuscript. If the question refers to our own annotation process, we note that no scoring normalisation is needed, as we reached consensus in plenary meetings on all instances where our annotations disagreed. Disagreements were resolved case by case by applying a consistent, agreed-upon interpretation of the call across the CAFs, which is reported in the methodological section. 

 Reviewer comment (Q6)

 Assuming that the scoring leads to a ranking of candidates, what does that lead to: e.g. invitation for an interview of the X highest scoring candidates, all candidates above a cut-off score, etc.?

 Response (R6)

 In the evaluation, each candidate is assigned a score for each CV section, consistent with the score partitioning reported in Table 2. In these specific calls, there was no threshold for admission to the interview. If a candidate's total score exceeds the relevant threshold reported in Table 2, they are declared eligible for career advancement and ranked accordingly on a ranked eligibility list (graduatoria). Being declared eligible does not, by itself, entail career progression, because only a limited number of positions are assigned to each call. However, the ranked eligibility list remains open even after the selection process is complete, as additional funds may become available later on, allowing further positions to be filled by progressively drawing from the list in order of score, without issuing a new competitive selection.  We included a concise explanation of this mechanism in the revised manuscript, and updated Figure 2 to make explicit the fact that the final step of the selection process is the publication of ranked eligibility lists by CNR. 

 Reviewer comment (Q7)

 Assuming that interviews are held, are the scores or rank order integrated in the final decision (career progression [promotion] or not)? And if so, how are score and interview impression combined?

 Response (R7)

 Scores from CV sections, seniority (when applicable), and oral examinations are combined additively, as specified in Table 2. The resulting total determines the final ranking in the eligibility list. The scores of the research products and qualifications portfolio, the narrative CV sections, seniority, and the oral exam (whenever required) are combined by adding up their scores, following the instructions reported in Table 2. The resulting total determines the final ranking of each candidate in the eligibility list, as described above in R6.

 Reviewer comment (Q8)

 Please explain better why readers should be interested in this Italian case history. My impression is that in international perspective such an amazingly granulated policy and procedure for career progression is quite rare. What can others learn from your in-depth exploration of this single case?

 Response (R8)

 Existing studies on CoARA/ARRA implementation focus mainly on institutional adoption at a policy level and rely on large-scale surveys to assess institutional policy adoption. However, these rarely examine how these commitments are translated into actual assessment criteria at the operational level. In contrast, this study study analyses how four ARRA commitments are operationalised in evaluation criteria. The granularity the reviewer notes is, in this respect, not an idiosyncrasy of the Italian case but a methodological asset: The CNR case provides a rare setting in which a common reform mandate is implemented across 90 independent committees, each responsible for defining the evaluation criteria in their respective CAF. This allows us to document implementation variance with a level of resolution that survey-based, cross-institutional studies cannot provide.  We also believe that this case study is relevant beyond Italy for RPOs in countries which have a similar evaluation system (such as Spain). What is comparatively unusual in the CNR case is not the devolution itself, but the fine-grained, quantitatively scored criteria produced by each of the 90 committees, which allowed us to systematically compare compliance with the ARRA commitments across many parallel implementations of the same reform mandate. We believe other large RPOs with similarly devolved evaluation structures, even where their own criteria are less granular than CNR's, may find the analytical framework we propose directly applicable for auditing their own practices against ARRA's commitments.

 Reviewer comment (Q9)

 Please openly recognize that you’re studying an intermediate outcome (how good do the assessment criteria reflect the policy commitments of CNR?). You are not studying the real endpoint: do the career progression decisions reflect the CNR adoption of the CoARA and ARRA commitments?

   Response (R9)

 We thank the reviewer for this important methodological observation and fully agree that it deserves explicit acknowledgement. The study does indeed measure an intermediate outcome: the extent to which the evaluation criteria formulated by committees reflect CNR's endorsement of ARRA commitments. We did not study the final endpoint, that is, whether actual career progression decisions reflect those commitments in practice, and this has been explicitly acknowledged in the Limitations section that we have added to the revised manuscript. However, we would like to respectfully push back on the reviewer's assumption that formal criteria play only a limited role in actual committee decisions, at least in the specific institutional and legal context of CNR.  In Italy, competitive public-sector selection procedures, including CNR's career-progression calls, are subject to administrative law and can be challenged by dissatisfied or unsuccessful candidates before administrative courts. Because of this, evaluation committees operate under a strong legal incentive to apply their own published criteria consistently and literally, particularly for Section I (selected products and qualifications).  Any departure from the published criteria, or any inconsistency in how point fractions are awarded across candidates, is a recognised ground for appeal. As a result, committees have comparatively little room for ad hoc discretion at the scoring stage, unlike the more loosely coupled relationship between formal criteria and final committee judgement that the reviewer describes from his own experience in other contexts. This legal framework also helps explain one of the patterns we observe in our analysis: entirely formulaic or bibliometric criteria are often viewed favourably in this specific context, precisely because they leave little room for subjective interpretation and are comparatively difficult to contest on appeal.  More qualitative or judgment-based criteria, despite being more aligned with ARRA commitments, can be perceived as exposing both candidates and committees to greater legal uncertainty. We believe this institutional incentive is one of the underlying drivers of the prevalence of mechanical, formula-based scoring documented in our analysis of Commitment 

 We note, however, that this tight coupling between criteria and outcomes is most relevant to the CV Section I. The narrative CV sections (II-IV) and, where applicable, the oral examination involve a greater degree of committee discretion, and we cannot make the same claim for those parts of the evaluation. The relationship between the criteria and the final outcomes in CV sections II-IV remains an important open question, which we have flagged as an interesting direction for future research, despite being subject to the availability of non-public committee deliberation records. 

 Reviewer comment (Q10)

 The criteria the CNR career progression committees used in 2023 were evaluated in this project. Please reflect on what has changed since. This might be important, because the culture change in researcher assessment seems to have accelerated substantially over the last few years.

 Response (R10)

 We would like to clarify that the 2023 competitive calls analysed in this study are the most recent ones available. No subsequent career advancement calls have been announced since then, meaning that the 2023 procedures represent the current state of the art at CNR.  We also note that the timeline between the completion of a competitive procedure and the availability of its documents for analysis is unfortunately long. This means that even if new procedures were announced in the near future, the materials would not be available for analysis for a considerable time thereafter.  We acknowledge that the culture of research assessment reform has accelerated substantially in recent years, and that future calls may reflect greater awareness of and alignment with ARRA commitments. A comparison with future calls would therefore be a valuable next step, and we highlighted this as both a limitation of the current study and a direction for future research in the revised manuscript.

 Reviewer comment (Q11)

 It is unacceptable that a study limitation section is missing.

 Response (R11) We agree with the reviewer and have added a dedicated limitations section in the revised manuscript. Summarising from the previous observations, the new section explicitly addresses:  (i) the focus on criteria as an intermediate outcome rather than actual career progression decisions;  (ii) the restriction to the 2023 calls, which represent only the earliest stage of ARRA implementation at CNR;  (iii) the unavailability of internal committee working procedures;  (iv) the potential subjectivity inherent in our qualitative annotation of criteria, which we mitigated through extensive collegial discussion among all four authors but cannot entirely eliminate.

# Reviewer report: Reviewer response for version 1

**DOI:** 10.21956/openreseurope.25286.r74924

**Published:** 2026-06-17

**Authors:** Lea Maria Ferguson

**Affiliations:** 1 Helmholtz Open Science Office, Helmholtz-Gemeinschaft:, Potsdam, Brandenburg, Germany

**1. Summary of article**

 The article presents an analysis of data gained from the 2023 competitive calls for career procedures at National Research Council of Italy (CNR) against the four core commitments of the Agreement on Reforming Research Assessment (ARRA) as proposed by the Coalition for Advancing Research Assessment (CoARA) and as supported by CNR in 2022 through its signature and membership. Based on an extensive analysis of more than 4000 evaluation criteria by 90 evaluation committees, the article concludes that significant gaps remain between institutional policy and actual practice.

**2. Presentation of work and engagement with literature**

- The article provides a clear and comprehensive overview and embedding into the current literature in the field. 
- The article distinguishes between “researchers” and “technologists”. As this distinction might not be universally understood across disciplines and the cited Italian document might not be accessible to all readers, the authors may wish to include a brief definition or description of the different roles or profiles entailed by these terms when they are first introduced. This might enhance readers’ understanding of the significance of the distinction of these roles concerning different aspects of the analysis: 1) especially for the different CAFs, and also as a reasoning for why the results are treated separately and not aggregately, and 2) particularly in terms of the different results discussed, such as better compliance with ARRA for some of evaluation procedures of technologists as compared to researchers.  

**3. Study design and technical soundness**

- Considering the duration of the 3-year reform approach (2023-2025), the choice for data collection and subsequent analysis from February to July 2025 and publication date in spring 2026 are timely and suitable, and they lend valuable insights into the noteworthy gap between policy and practice at CNR. 
- By signing ARRA, CNR entered into the following commitments: 1) to publish an “Action Plan with defined milestones within one year of signing the agreement”, 2) to regularly demonstrate “progress towards reviewing, developing and evaluating criteria, tools and processes that fulfil the core commitments, with a touch point at end of 2027 or within five years of signing the agreement”. Regarding this time frame, the article’s analyzed time-period seems early and fruitful further insights into the reform initiative(s) might be gained from also investigating the full five-year timeframe.

**4. Details of methods and analysis**

- The analysis presents an impressive approach towards qualitative and quantitative criteria of research assessment (and their distinction) that provides valuable insights into ongoing research assessment procedures. 

**5. Availability of source data and materials**

- This detailed and well-documented analysis (openly available under CC BY via Zenodo) lends itself not only to gain insights into the Italian research landscape but might also be adapted to other (national) contexts and analyses of research assessment developments.
- To ensure the full extent of reusability, machine-readability, and long-term preservation, the source data might additionally be provided in CSV format. 

**6. Conclusions supported by results**

- The identified gaps between ARRA’s four core commitments and the practical and institutional reality of research assessment in its implementation during the 2023 competitive calls for career procedures at CNR are well analyzed, documented, presented, and explained. These conclusions allow for a critical look at the status-quo as well as paving the way for more transformative and far-reaching future reforms. 
- In some parts of the conclusions, the language employed is somewhat harsh (using variations of “fail”, “infringe”, “breach”, and “violate”). This framing might be softened somewhat, considering the following: 1) The institutions signing ARRA do so willingly; calling out these institutions for “failing” places a heavy burden on the same institutions actively participating in the reform movement (while some institutions are not even participating yet). 2) Regarding the dense timeframe of the beginnings of ARRA/CoARA and CNR’s reform approach in 2022-2023, the element of time/timing might also be taken into further consideration. 3) The employed language implies negative (perhaps even) legal repercussions; this seems to go somewhat beyond the commitment that institutions agree to by becoming a signatory of ARRA and member of CoARA. 

If applicable, is the statistical analysis and its interpretation appropriate?

I cannot comment. A qualified statistician is required.

Is the study design appropriate and is the work technically sound?

Yes

Is the work clearly and accurately presented and does it engage with the current literature?

Yes

Are the conclusions drawn adequately supported by the results?

Yes

Are sufficient details of methods and analysis provided to allow replication by others?

Yes

Are all the source data and materials underlying the results available?

Yes

Reviewer Expertise:

I have been working in the fields of open science, open access and research policy for the last 8 years, with a focus on monitoring open science, implementing open access, and reforming research assessment.

I confirm that I have read this submission and believe that I have an appropriate level of expertise to confirm that it is of an acceptable scientific standard.

# Response

**Published:** 2026-07-03

**Authors:** Lottie Provost

**Affiliations:** Istituto di Linguistica Computazionale “A. Zampolli”, Consiglio Nazionale delle Ricerche, Italy

We thank the Reviewer for their positive assessment of our work, we address the comments point by point below.

 1. Definition of "researchers" and "technologists"

 We agree that this distinction may not be immediately clear to international readers. We have edited the manuscript with a footnote clarifying that in the Italian public research sector, "researchers" (ricercatori) are staff whose primary role is scientific investigation, while "technologists" (tecnologi) are staff whose work focuses on the development, management and valorisation of technologies and research infrastructures, although in practice the boundaries can overlap. 

 2. CSV format for source data

 We have provided the underlying datasets in CSV format in addition to the existing XLSX files on Zenodo, to enhance machine-readability, reusability, and long-term preservation. These can be found in the Underlying data section of the revised manuscript.

 3. Language Regarding the use of harsh language,

 we wish to note that in the CNR case, the boundary between voluntary commitment and legal obligation is less straightforward than in other ARRA signatory contexts. The ARRA commitments were explicitly incorporated into the regulatory documents governing the calls, which carry legal value under Italian administrative law. We deem this specificity of the CNR case to be worth retaining in the discussion.
